# RNA Microarray Analysis in Prenatal Mouse Cochlea Reveals Novel IGF-I Target Genes: Implication of MEF2 and FOXM1 Transcription Factors

**DOI:** 10.1371/journal.pone.0008699

**Published:** 2010-01-25

**Authors:** Hortensia Sanchez-Calderon, Lourdes Rodriguez-de la Rosa, Marta Milo, Jose G. Pichel, Matthew Holley, Isabel Varela-Nieto

**Affiliations:** 1 Instituto de Investigaciones Biomedicas “Alberto Sols”, CSIC-UAM, Madrid, Spain; 2 Unit 761, Centro de Investigación Biomédica en Red de Enfermedades Raras (CIBERER), Unit 761, Instituto de Salud Carlos III, Madrid, Spain; 3 Department of Biomedical Science, University of Sheffield, Sheffield, United Kingdom; 4 Centro de Investigacion del Cancer, Instituto de Biologia Molecular y Celular del Cancer, CSIC/Universidad de Salamanca, Salamanca, Spain; Universidade Federal do Rio de Janeiro, Brazil

## Abstract

**Background:**

Insulin-like growth factor-I (IGF-I) provides pivotal cell survival and differentiation signals during inner ear development throughout evolution. Homozygous mutations of human *IGF1* cause syndromic sensorineural deafness, decreased intrauterine and postnatal growth rates, and mental retardation. In the mouse, deficits in IGF-I result in profound hearing loss associated with reduced survival, differentiation and maturation of auditory neurons. Nevertheless, little is known about the molecular basis of IGF-I activity in hearing and deafness.

**Methodology/Principal Findings:**

A combination of quantitative RT-PCR, subcellular fractionation and Western blotting, along with *in situ* hybridization studies show IGF-I and its high affinity receptor to be strongly expressed in the embryonic and postnatal mouse cochlea. The expression of both proteins decreases after birth and in the cochlea of E18.5 embryonic *Igf1^−/−^* null mice, the balance of the main IGF related signalling pathways is altered, with lower activation of Akt and ERK1/2 and stronger activation of p38 kinase. By comparing the *Igf1^−/−^* and *Igf1^+/+^* transcriptomes in E18.5 mouse cochleae using RNA microchips and validating their results, we demonstrate the up-regulation of the FoxM1 transcription factor and the misexpression of the neural progenitor transcription factors *Six6* and *Mash1* associated with the loss of IGF-I. Parallel, *in silico* promoter analysis of the genes modulated in conjunction with the loss of IGF-I revealed the possible involvement of MEF2 in cochlear development. E18.5 *Igf1^+/+^* mouse auditory ganglion neurons showed intense MEF2A and MEF2D nuclear staining and MEF2A was also evident in the organ of Corti. At P15, MEF2A and MEF2D expression were shown in neurons and sensory cells. In the absence of IGF-I, nuclear levels of MEF2 were diminished, indicating less transcriptional MEF2 activity. By contrast, there was an increase in the nuclear accumulation of FoxM1 and a corresponding decrease in the nuclear cyclin-dependent kinase inhibitor p27^Kip1^.

**Conclusions/Significance:**

We have defined the spatiotemporal expression of elements involved in IGF signalling during inner ear development and reveal novel regulatory mechanisms that are modulated by IGF-I in promoting sensory cell and neural survival and differentiation. These data will help us to understand the molecular bases of human sensorineural deafness associated to deficits in IGF-I.

## Introduction

Insulin-like growth factor I (IGF-I) is a member of the insulin family that regulates development and tissue homeostasis [1], [2]. It acts primarily by binding with high affinity to the IGF-I tyrosine kinase receptor (IGF1R) and its activity is modulated by IGF-binding proteins (IGFBP) [3]. IGF factors, receptors and binding proteins form the IGF system. Peak expression of IGF-I in the nervous system occurs during late embryonic and neonatal periods, although relatively high expression is maintained in areas of high plasticity, such as the olfactory bulb and hippocampus [4]. Mutations in mice have shown that IGF-I modulates survival, proliferation and differentiation of all the neural lineages studied, and it promotes synaptogenesis and dendritic arborisation in projection neurons [4], [5]. Activation of the IGF1R leads to phosphorylation of insulin-receptor-substrates and activation of the cytosolic serine-threonine MAP kinases and Akt kinases that induce the translocation of transcription factors to the cell nucleus, thereby initiating specific gene expression programmes [6], [7]. Deficits in IGF-I are associated with severe nervous system disorders, including neurodegenerative diseases, and treatment with IGF-I promotes neural cell repair and regeneration [8]. Homozygous mutations in human *IGF1* result in a wide range of disorders including intrauterine growth retardation, postnatal growth failure, microcephaly and mental retardation. They also cause severe bilateral sensorineural deafness (ORPHA73272; http://www.orpha.net; [9], [10], [11]).

Normal development of the inner ear depends on IGF-I signalling [12]. The auditory sensory epithelium is the organ of Corti, which is composed of linear rows of hair cells and supporting cells housed within the cochlea. The mouse inner ear develops from embryonic day (E)8 from the otic placode, a patch of ectoderm that invaginates and pinches off to form the otic vesicle from which all the sensory epithelial cells and sensory neurons are derived. By E15.5, the organ of Corti has acquired its full complement of cell types although it does not become functionally mature until the onset of hearing at postnatal day 12–14 [13]. *Igf1* null (*Igf1^−/−^*) mice are dwarfs that present organ-specific growth retardation and a 30% reduction in brain size. The impact on the nervous system includes loss of selective neuronal populations, hypomyelinization and reduced peripheral conduction velocities [14], [15]. As in man, IGF-I deficit in the mouse causes all-frequency bilateral sensorineural hearing loss and a delayed response to acoustic stimuli [16]. From postnatal day P5 cochlear development is severely impaired in mutant *Igf1^−/−^* mice, which develop a smaller cochlea with an immature tectorial membrane. In addition, these animals suffer aberrant synaptogenesis, abnormal innervation of the sensory hair cells in the organ of Corti, poor myelination and a significant decrease in the number and size of auditory neurons [17], [18]. The marked reduction reported in neural cell number at P20 is due to increased apoptotic cell death of both neurons and Schwann cells [17].

Here, we have explored the otic-specific targets of IGF-I signalling to further understand the function of this factor in the inner ear and how its deficit causes neurosensorial deafness. Comparative gene expression profiles from the cochlea of wild-type (*Igf1^+/+^*) and *Igf1* ^−/−^ mice at embryonic day E18.5 suggest that IGF-I modulates sensory cell differentiation and neural cell fate decisions during late otic development. The expression patterns of *Six6*, *Mash1* and *Fgf15* are altered in the cochleae of *Igf1^−/−^* mice. Changes also occur in the expression, protein levels and nuclear localization of *FoxM1*, a forkhead box transcription factor that is ubiquitously expressed in proliferating cells and one of its targets the cyclin-dependent kinase inhibitor p27^Kip^. *In silico* analysis of the promoter regions of differentially expressed genes selected from the microarray analysis of null versus wild type cochleae at E18.5, pointed to the transcription factor myocyte enhancing factor 2 (MEF2) as a novel downstream target of cochlear IGF-I signalling. The nuclear expression of MEF2A and D was lower in the absence of IGF-I. Thus, for the first time we show that MEF2 and FoxM1 activities are modulated by IGF-I in the mouse cochlea. These results also provide novel clues to the molecular mechanisms underlying otic development and the causes of neurosensorial deafness associated with defects in IGF-I.

## Materials and Methods

### Mouse Handling and Genotyping

Heterozygous mice in which the *Igf1* gene underwent targeted disruption were bred, maintained and genotyped as described [14], [17]. In brief, E18.5 *Igf1^−/−^* embryos on a C57BL/6J genetic background, which die at birth, were used for the DNA array study, whereas a hybrid MF1/129/sv genetic background was used to increase survival in the resultant null mice as described [14], [17]. Adult survival was around 20% of the newborn null *Igf1* ^−/−^ mice. Both mouse strains showed similar cochlear gene and protein expression profiles when tested at E15.5 and E18.5. Mouse genotypes were identified using the REDExtract-N-Amp™Tissue PCR Kit (XNAT, Sigma) following the manufacturer's instructions and with the following primer sets specific for the *Igf1* and neomycin genes (*Igf1* forward 5′-GTCTAACACCAGCCCATTCTGATT-3′; *Igf1* reverse 5′-GACTCGATTTCACCCACTC-GATCG-3′; neomycin forward 5′-GCTTGGGTGGAGAGGCTAT-CC-3′; and neomycin reverse 5′-CCAGCTCTTCAGCAATATCACGGG-3′). Hearing was tested in adult animals by recording auditory brainstem responses as described [16] (results not shown). Animals were humanely sacrificed and all procedures were in accordance with the European Council Directive (86/609/EEC) and the Bioethics Committee of the CSIC.

### Transcriptome Analysis by GeneChip Arrays

E18.5 was selected because most *Igf1^−/−^* mice die in early postnatal development. E18.5 cochleae from two *Igf1^+/+^* and two *Igf1^−/−^* embryos were dissected and pooled to obtain RNA. Three independent RNA pools of each genotype (6 mice) were isolated with Trizol (Invitrogen) following the manufacturer's instructions. The purity of RNA was assessed with an Agilent Bioanalyzer 2100 (Agilent Technologies). Six additional microarrays were hybridized with whole lung RNA obtained from the same mice or their siblings and were included in the analysis to compare the expression profiles of different organs (GSE17157; JGP manuscript in preparation).

Cochlear complementary RNA (cRNA) for hybridization to MOE430A Genechips® (Affymetrix) was prepared by sequentially generating cDNA with the one-Cycle cDNA Synthesis Kit, which was purified and used as a template in the in vitro transcription reaction for cRNA amplification and biotin labelling. The cRNA was then hybridized to the GeneChip® arrays and scanned with a GeneChip® Scanner 3000 7G 4C (Affymetrix).

An initial analysis was performed with MAS5.0 (Affymetrix) and Robust Multiarray Average (RMA) software [19], which indicated a very high variability that was associated with the biological variability and non-specific hybridization. Because most of the tissue-specific genes are expressed at low levels at this developmental stage, the high background signal of common genes generated a very high noise-to-signal ratio. The software package PUMA (Propagating Uncertainty in Microarray Analysis) was then used to estimate the gene expression levels. This package, integrated in the Bioconductor project (http://www.bioconductor.org), uses novel probabilistic models to analyse affymetrix GeneChip array data. Specifically, the multi-mgMOS (multi-chip modified gamma model for oligonucleotide signal) model was used to extract gene expression levels and their estimated uncertainties [20]. The analysis of the Fold change (FC) was used in combination with the Probability of Positive Log Ratio (PPLR) algorithm from the PUMA package, to reduce the number of false positives [21]. PPLR associates probability values (between 0 and 1) to each log ratio, which represents the probability of the log Ratio being positive. This probability is a measure of the false positive detection of differential expression and it allows the selected Differentially Expressed (DE) genes to be ranked in order of the robustness of the prediction. We defined DE genes in *Igf1^+/+^* mice as those that presented a positive FC greater than one log_2_ unit with an associated probability higher than 0.95. Conversely, in *Igf1^−/−^* mice we defined DE genes as those that presented a negative FC less than -1 with an associated probability lower than 0.05.

Genes were classified by their ontology and by the biological processes in which they are implicated using the PANTHER Classifications Systems software (http://www.pantherdb.org) and FATIGO+ (http://babelomics.bioinfo.cipf.es/).

### Transcription Factor Analysis

The promoter regions of up-regulated genes in the *Igf1^−/−^* mouse cochlea selected either by their DE ranking and/or their association with sensorial deficits in humans (*IL-13ra1*, *Fgf15*, *Foxm1*, *Six6*, *Rorb*, *Rp1h* and *Ush1c*), were compared using the MEME software (http://meme.sdsc.edu/meme/intro.html) to identify common motifs. The promoter regions were selected using the PromoSer database (http://biowulf.bu.edu/zlab/PromoSer/) extracting 1.5 Kb upstream and 50 bp downstream of the Transcription Start Site (TSS). Only motifs with a pair-wise correlation lower than 0.30 were selected and the selected motifs were searched for known transcription factor binding sites using TESS (http://www.cbil.upenn.edu/cgi-bin/tess/). Only the transcription factors sites with the highest Log-likelihood score were selected. Similarly, down-regulated genes with a FC>1 were grouped and the common motifs in the promoter region were analysed using either the method indicated above or the FATIGO+ software with similar results.

### Low Density Arrays and Quantitative RT-PCR

TaqMan® Low Density Arrays containing three replica probes for each of the twenty genes selected from the array data were hybridized with cDNA generated by reverse transcription (High Capacity cDNA Reverse Transcription Kit. Applied Biosystems). cDNA was prepared from three to five different RNA pools corresponding to six to ten mice for each genotype. Each RNA pool was isolated as described above from the pooled cochleae from two embryos or mice for each genotype taken at the following times: E15.5, E18.5, P5, P15, P30, P60 and P90. PCR was performed on an Applied Biosystems 7900HT Fast Real-Time PCR System and the genes were selected on the basis of their FC, physiological interest and the availability of appropriate TaqMan® probes. In addition, probes to test the temporal expression of the IGF system factors, receptors and transport proteins were used. Eukaryotic 18S rRNA was chosen as an endogenous housekeeping control gene and the estimated gene expression was calculated as 2^−ΔCt^, multiplying this value by a factor of 10^6^ to generate a clearer graphical representation. Alternatively, gene expression was analyzed by real time PCR using validated probes from TaqMan® Gene Expression Assays (https://products.appliedbiosystems.com/ab/en/US/adirect/ab?cmd=ABGEKeywordSearch; Applied Biosystems). Probes used are listed in Table S1 and included those for *Igf2*, *Irs2*, *Foxm1*, *Foxg1*, *Mash1*, *Mef2a*, *Mef2c and Mef2d*. Assays were done following manufacture's instructions and using as reference the expression levels of 18S. The relative quantification values (RQ) were calculated by the 2^−ΔΔCt^ method and data are presented as means of log_10_RQ.

### *In Situ* Hybridization

*In situ* hybridization was performed essentially as described previously [22], with minor modifications. The cDNA used to generate the *in situ* hybridization probes are detailed in Table S2 Three E15.5, E18.5 and P5 mice per genotype were tested in parallel in three independent experiments. No signal was obtained with the control sense probes (data not shown). Sections were incubated overnight at 70°C with 1 µg/ml of the digoxigenin-labeled probes, and binding detected by overnight incubation with alkaline phosphatase-conjugated anti-digoxigenin antibody (1∶3500, Roche), which was visualised with NBT (Nitro blue tetrazolium chloride)/BCIP (5-Bromo-4-chloro-3-indolyl phosphate, toluidine salt; 1∶50, Roche) or Fast Red (Roche) for fluorescence.

### Cochlear Morphology and Immunohistochemistry

Selected E15.5, E18.5, P5 and P15 cochlear sections were examined by dual *in situ* hybridization and immunohistochemistry as described in [23] using the primary antibodies summarized in Table S3. Sections were then sequentially covered with the secondary antibody solution (1∶100, biotin-conjugated anti-mouse IgG or biotin-conjugated anti-rabbit, Chemicon), and extravidin peroxidase (1∶200, Sigma). Finally, antibody binding was visualised using DAB as the chromogen and the sections mounted in Mowiol for observation under a Nikon 90i microscope. When indicated, Alexa Fluor 488 goat anti-rabbit, Alexa Fluor 546 goat anti-rabbit, Alexa goat anti-mouse 488 or Alexa donkey anti-goat 488 dyes (1∶400, Molecular Probes) were used as the secondary antibody. Three embryos or mice per genotype were tested in parallel in three independent experiments. Control experiments without primary antibody were carried out for each reaction and indicated that the staining pattern was specific for antigen recognition (data not shown).

### Cochlear Protein Extraction and Analysis

Frozen cochleae from E15.5, E18.5, P5, P15, P60 and P90 mice were pooled and homogenized in 200 µl of ice-cold RIPA lysis buffer containing 0.01% of the P8340 protease and P5726 phosphatase inhibitor cocktails (Sigma) and heated to 95°C for 5 min. Cochlear extracts were cleared by centrifugation at 11,800 rpm for 5 min at 4°C, and the supernatant was stored at −70°C until use. Three to six different pools from each genotype were used. When indicated, NE-PER® Nuclear and Cytoplasmic Extraction Reagent (PIERCE Biothecnology) was used to prepare the cytoplasmic and nuclear extracts from E18.5 and P15 cochleae as indicated by the manufacturers. The protein content of the samples was determined with the Coomassie® Plus Protein Assay Reagent Kit or Micro BCA Protein Assay Kit (PIERCE Biothecnology) using BSA as the standard.

Equal amounts of cochlear protein were subjected to SDS-PAGE on 8%, 10% or 15% polyacrylamide gels and the proteins were then transferred to PVDF membranes in a Bio-Rad Trans Blot apparatus according to the manufacturer's instructions. After incubation with a blocking solution, the membranes were probed overnight at 4°C with the appropriate primary antibodies summarized in Table S3. All antibodies were diluted in blocking solution except those against Akt, P44/42 ERK and p38 MAPK, which were diluted in TBS-T containing 5% BSA. The membranes were then washed and incubated with the appropriate peroxidase conjugated secondary antibodies for 1 h at RT. Immunoreactive bands were visualized by ECL (GE Healthcare Amersham) and the bands were quantified by densitometry with NIH Image J software. Statistical significance was estimated by Student's t-test after using Levene's test to confirm the equality of variances.

## Results

### Spatiotemporal Pattern of Expression of the IGF System Elements and Modulation of Target Kinase Activities in the *Igf1^−/−^* Null Mouse

**Figure 1:**
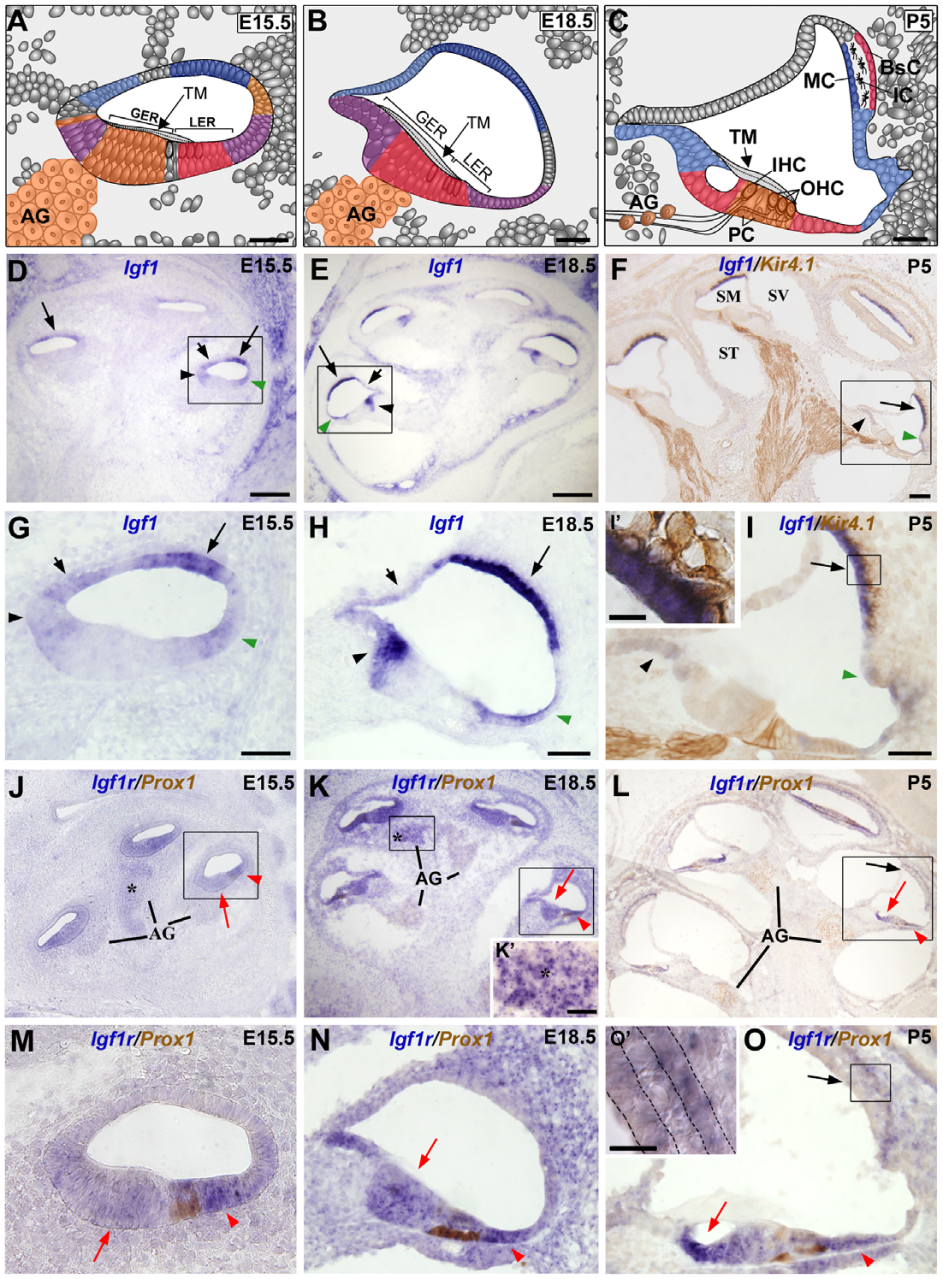
*Igf1* and *Igf1r* mRNA expression in the cochlea. (**A–C**) Cartoons of the organ of Corti at E15.5, E18.5 and P5 show the expression of *Igf1* (blue), *Igf1r* (orange and red) or both (purple). (**D–O**) *In situ* hybridization of *Igf1* (D-I,I') and *Igf1r* (J-O, O') in normal embryos at E15.5 (D,G,J,M), E18.5 (E,H,K,N) and in P5 mice (F,I,L,O). Dual immunostaining with anti-Kir4.1 was performed to identify the neural projections, the stria vascularis and pillar cells (F,I,I') and with anti-Prox1 to identify the pillar cells, Deiter's cells, and auditory neurons (J–O,O'). (**D-I**) *Igf1* expression was located in the stria vascularis (long black arrows), spiral limbus (black arrowheads), outer sulcus (green arrowheads) and Reissner's membrane (short black arrow). At P5 the *Igf1* expression in the stria vascularis was observed in the marginal cells (I'). (**J,K,M,N**) At E15.5 and E18.5, *Igf1r* was strongly expressed in the GER (red arrow) and weakly in the LER (red arrowhead). (**L,O**) At P5, *Igf1r* expression presented a complementary pattern to that of *Igf1* and was observed within the inner spiral sulcus (red arrows), Claudius and Hensen's cells (red arrowheads). *Igf1r* was also located in the AG (asterisk in J,K, K') and in the basal cells of the stria vascularis (O'). Three embryos per genotype were tested in parallel in three independent experiments. GER, greater epithelial ridge; IHC, inner hair cells; LER, lesser epithelial ridge; OHC, outer hair cells; PC, pillar cells; AG, auditory ganglion; SM, scale media; ST, scala tympani; SV, scala vestibuli; TM, tectorial membrane. Scale Bars: D,E,F, 150 µm (D,E,F,J,K,L); A,B,C, 50 µm; G,H,I, 50 µm (G,H,I,M,N,O); I', 10 µm; O', 20 µm and K', 30 µm.

Previous studies have shown that cochlear structures are positive for IGF-I immunostaining, which was observed in the stria vascularis, spiral limbus and sensory supporting cells, as well as in subpopulations of auditory ganglion neurons at postnatal day P20 [17]. However, because IGF-I is a hormone secreted by the liver, it was important to determine whether or not it was synthesised in the cochlea. To address this question we performed *in situ* hybridization for *Igf1* and *Igf1r* at stages E15.5, E18.5 and P5 (Fig. 1).

At stages E15.5 and E18.5, the auditory epithelium can be divided into the greater epithelial ridge (GER), which includes the single row of inner hair cells, and the lesser epithelial ridge (LER), which includes the three rows of outer hair cells (Fig. 1A–B). By P5 (Fig. 1C), the structure of the organ of Corti more closely resembles that of the adult. *Igf1* and *Igf1r* temporal expression patterns are shown in Fig. 1A–C painted in blue and red tones, respectively. At E15.5, *Igf1* mRNA was strongly expressed in an area corresponding to the future stria vascularis and more weakly in those areas that will give rise to Reissner's membrane, the spiral limbus and the outer sulcus (Fig. 1D,G). The expression of Prox1 was used to define the LER (see Fig. 1M; [24]). At E18.5, *Igf1* was still expressed strongly in the stria vascularis and spiral limbus, while it was relatively weak in the outer sulcus and Reissner's membrane (Fig. 1E,H). The expression of *Igf1* was restricted by P5, when it was detected in the marginal cells of the stria vascularis and it overlapped with the cells expressing the Kir4.1 potassium channel (Fig. 1F,I,I'). Weaker expression was observed in the inner and outer sulcus.

*Igf1r* was ubiquitously expressed at E15.5 but it was stronger in the GER, LER and auditory ganglion (Fig. 1J,M). The expression pattern remained similar at E18.5 (Fig. 1K,N), although it was notably stronger in the apical turn of the auditory ganglion (Fig. 1K'). As with *Igf1*, the expression of *Igf1r* was more restricted by stage P5 and interestingly, the pattern was complementary to that of *Igf1* with this receptor being confined to the inner spiral sulcus, Hensen's, Claudius cells and the basal cells of the stria vascularis (Fig. 1L,O,O'). The pattern of expression of the *Igf1r* did not show any change in the *Igf1^−/−^* null mouse cochlea with respect to the wild type cochlea at the stages studied (E15.5, E18.5, P5 and P15; Fig. S1).

**Figure 2:**
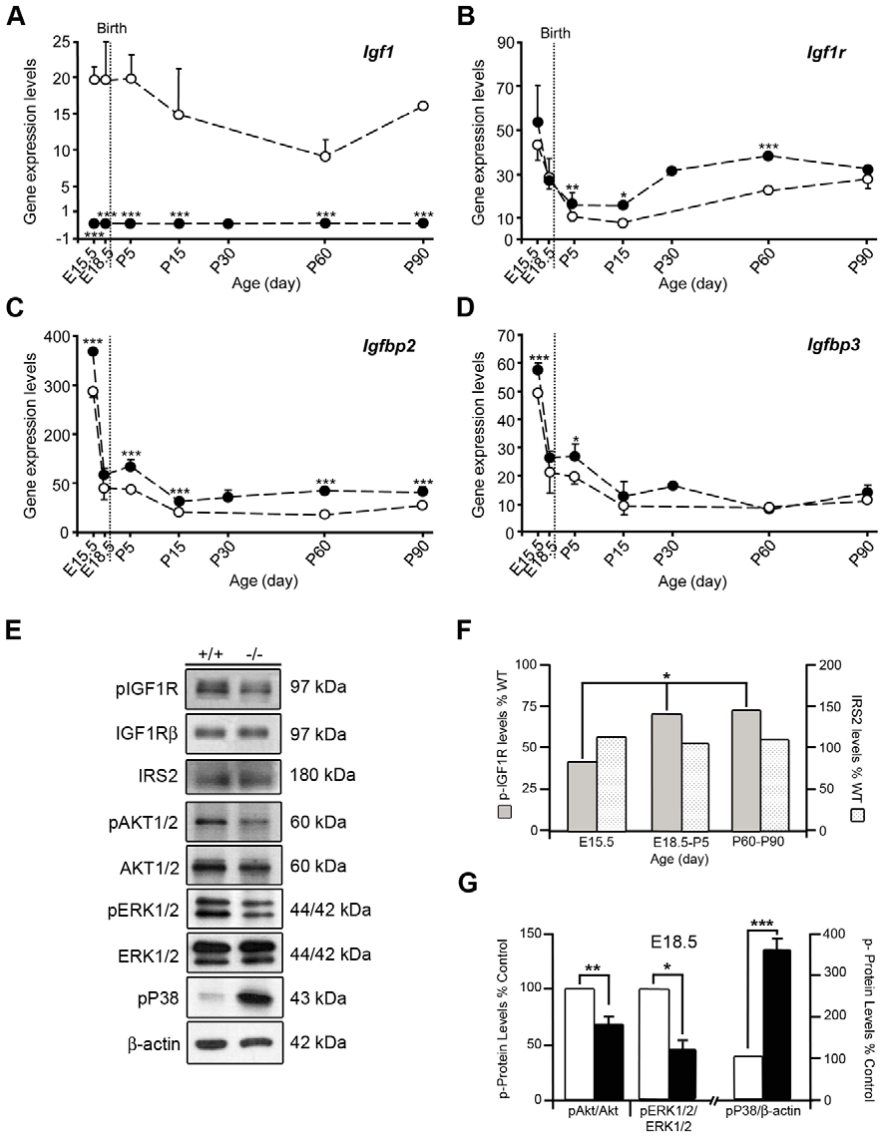
Time-course of mRNA expression of IGF-system genes and the activation levels of signalling mediators in the E18.8 cochlea. (**A–D**) mRNA expression levels of *Igf1*, *Igf1r*, *Igfbp2* and *Igfbp3* were analyzed by qRT-PCR in *Igf1^+/+^* (open circles) and *Igf1^−/−^* (closed circles) mice at E15.5 and E18.5 (n = 8), P5, P15, P30, P60 and P90 (n = 6). Eukaryotic 18S rRNA was used as the endogenous housekeeping control gene. The estimated gene expression was calculated as 2^−ΔCt^·10^6^. (**A**) *Igf1* expression was high in normal cochlea and absent in the null mice. (**B**) *Igf1r* expression in normal cochleae decreased dramatically from E15.5 to P5 and increased with age thereafter. In the *Igf1^−/−^* cochlea, *Igf1r* followed the same pattern but consistently presented higher levels at all time points studied. *Igfbp2* (**C**) and *Igfbp3* (**D**) mRNA levels were high at E15.5 but they dropped thereafter. Their profiles were slightly higher in the *Igf1^−/−^* cochlea. (**E**) IGF-I modulates IGF1R, ERK, Akt and p38 activation at E18.5. (**F**) Levels of phosphorylated-IGF1R and IRS2 in cochlear protein extracts from *Igf1^+/+^* and *Igf1^−/−^* mice were studied by Western blotting at E15.5, E18.5, P5, P60 and P90. Data are presented as percentage of *Igf1^−/−^* null mouse protein levels compared to the *Igf1^+/+^*. (**G**) To determine the levels of phosphorylated Akt^Ser473^, ERK and p38 MAPK, cochlear protein extracts from E18.5 *Igf1^+/+^* and *Igf1^−/−^* mice were analysed by immunoblotting. Membranes were re-probed with β-Actin as a loading control, and for the non-phosphorylated forms of AKT and ERK1/2. Films were scanned, densitometry performed by using ImageJ software and the levels were normalised by giving a value of 100 to the *Igf1^+/+^* mouse samples. Values are presented as mean±SEM of at least 3 different experiments involving at least 6 mice per condition for Akt, ERK and p38 MAPK. The statistical significance estimated by Student's t-test was as follows ***p<0.005; **p<0.01; *p<0.05.

The temporal expression profiles of several genes of the IGF system were studied in the cochleae of *Igf1^+/+^* and *Igf1^−/−^* mice by qRT-PCR. These included *Igf1*, *Igf1r*, *Ins2*, *Igf2*, *Igfbp2* and *Igfbp3* at stages E15.5, E18.5, P5, P15, P30, P60 and P90. In *Igf1^+/+^* mice the expression of *Igf1* remained high during development, despite the modest postnatal decrease, while as expected it was absent in the *Igf1^−/−^* cochlea (Fig. 2A). *Ins2* was not detected in wild type or mutant mice, at any of the time points studied (data not shown). In contrast, *Igf2* expression remained high during development and dropped after birth, the expression levels of *Igf2* did not show statistically significant differences between wild type and mutant mice at any of the time points studied (data not shown). Expression of *Igf1r* in the *Igf1^+/+^* cochlea decreased dramatically from E15.5 to P5, and it increased with age thereafter (Fig. 2B). In the *Igf1^−/−^* cochlea, *Igf1r* was expressed at higher levels than normal after birth and it remained proportionally higher throughout the period studied (Fig. 2B). IGFBP expression has been reported in the cochleae of several species [25], [26]. High levels of *Igfbp2* and *Igfbp3* expression were detected at E15.5, although this expression diminished rapidly thereafter. There was slightly higher expression of these binding proteins in the cochleae of *Igf1^−/−^* null mice (Fig. 2C–D).

Upon IGF-I binding, its high affinity receptor IGF1R tyrosine kinase activity is turned on and autophosphorylates receptor residues that act as docking sites for adaptor proteins like the insulin receptor substrate 2 (IRS2) [27], which in turn will activate downstream signalling pathways. Fig. 2E and Fig. S2 show that IGF1R is less phosphorylated in the *Igf1^−/−^* null mouse cochlea than in the wild type. Interestingly, there was a slight (30% p<0.05) increase in the tyrosine phosphorylation ratio of the IGF1R in the *Igf1^−/−^* from E18.5 to P90 when compared with the relative tyrosine phosphorylation ratio observed at E15.5; no changes could be shown for IRS2 levels at the times studied (Fig. 2F and S2).

IGF-I signalling is mediated by a network of intracellular mediators that include the phosphatidylinositol-3-kinase/Akt pathway and the mitogen-activated kinase cascades. In the E18.5 *Igf1^−/−^* null mouse cochlea there were reductions in the relative levels of activated phospho-Akt^Ser473^ (31%, p<0.01) and phospho-ERK1/2 (56%, p<0.05), whereas phospho-p38 MAPK was strongly activated (261%, p<0.005) when compared with the *Igf1^+/+^* wild type mouse cochlea (Fig. 2E,G). Akt and ERK1/2 MAPK activation are essential for cell survival and proliferation, whereas p38 MAPK forms part of the cellular response to environmental stress, such as ultraviolet light, heat, osmotic shock and inflammatory cytokines [28]. These data indicate that the IGF-I deficit at E18.5 diminishes the activity of the pathways that control cell proliferation and survival, whilst those involved in the cellular response to stress are heightened.

To recap, the main elements of the IGF system are present during the development of the cochlea and they are expressed in specific spatiotemporal patterns. IGF-I deficit affects the expression levels of the IGF system elements, and key IGF-I-activated signalling pathways are profoundly altered. These data, together with the reported morphological alterations and the profound sensorineural deafness that the deficit in IGF-I causes in mice and humans, prompted us to further study the molecular mechanisms underlying IGF-I activity in the developing mouse cochlea.

### Identification of Differentially-Expressed Genes in the E18.5 *Igf1^−/−^* Mouse Cochlea

To study the otic-specific gene targets of IGF-I, mRNA from whole cochleae of E18.5 *Igf1^+/+^* and *Igf1^−/−^* mice was hybridized with mouse ‘whole genome’ arrays (MOE430A) from Affymetrix. The results were submitted to the Gene Expression Omnibus (http://www.ncbi.nlm.nih.gov/geo/) with the accession number GSE11821.

We used multi-mgMOS and the model developed by the PUMA group to estimate gene expression levels with credibility intervals that quantify the measured variance associated with the estimated target concentration within a sample. This within-sample variance is a significant source of uncertainty in oligonucleotide arrays, especially for genes expressed at low levels. Final targets were selected using the PPLR algorithm that reduces the number of false positives. Genes that presented a Fold Change higher than a log_2_ unit of 1 (FC<1 with P<0.05 and FC>1 with P>0.95) were selected for further analysis. Following these criteria, the expression of 167 genes was seen to be considerably lower and 64 genes were expressed more strongly in the absence of IGF-I. These genes were then compared with the NCBI *Mus musculus* gene database, and ordered by gene ontology and biological processes with the programs PANTHER and FATIGO+. This analysis identified the different biological processes and cellular activities of the cochlear genes affected by IGF-I deficit (Fig. S3). A further selection was carried out on the basis of biological function, reported expression in the inner ear or association with human deafness, as well as for technical parameters as the FC and the low-variance between arrays. Table S4 shows selected genes differentially expressed in the absence of IGF-I, which include genes related to sensorial defects (*Ush1c*, *Esrrb* and *Tub*), ion transporters (*Cacna1f*, *Kcnd2*, *Kcnmb1* and *Mlc1*), the acetylcholine transporters *Slc18a3* and *Slc5a7* and the strial functional modulators *Esrrb* and *Cldn18*, among others that have been previously reported as cochlear-expressed genes.

To assess the organ specificity of the IGF-I target genes identified in this study, a parallel study was carried out on total RNA obtained from the lungs of *Igf1^−/−^* and wild type littermates (GSE17157; JGP, manuscript in preparation). The comparison of the differentially expressed genes obtained from both studies indicated that 94 genes were up-regulated in the lung, whereas only 64 genes were up-regulated in the cochlea. Interestingly none of these genes was common to the two tissues. In addition, 56 lung genes were down-regulated in the *Igf1^−/−^* mouse, in striking contrast to the weaker expression of 167 cochlear genes when compared to that in the wild type mouse cochlea. Only 3 genes were present in both databases: integrin alpha V (*Itgav*), solute carrier family 4 member 1 (anion exchanger - *Slc4a1*) and the ubiquitin specific peptidase 12 (*Usp12*).

The changes in the expression of 15 genes were confirmed by qRT-PCR using TaqMan probes where available, or by in situ hybridization. qRT-PCR has proven to be an efficient method to verify DNA array results and the predicted differences were confirmed for 68% of the genes studied (see Table S3). At E18.5, qRT-PCR of total cochlear mRNA confirmed that *Akr1c13*, *Fgf15*, *Foxm1*, *Mash1*, *Rp1h*, *Six6* and *Ush1c* transcripts were more strongly expressed in the cochleae of *Igf1^−/−^* mice [29], [30], [31], [32]. In contrast, *Foxg1*, which is involved in the morphogenesis of the mammalian inner ear [33], did not present a differential expression in the null mice (see Table S3).

These data contribute to our understanding of the molecular basis of the delayed maturation of the sensory epithelium reported in IGF-I deficit [17], extending the actions of this factor and highlighting a relationship with the Usher' syndrome molecules *Ush1c* [32], *Rp1h* [34] and *Tub* [35] whose mutations cause both deafness and blindness in men (ORPHA120433, ORPHA886).

**Figure 3:**
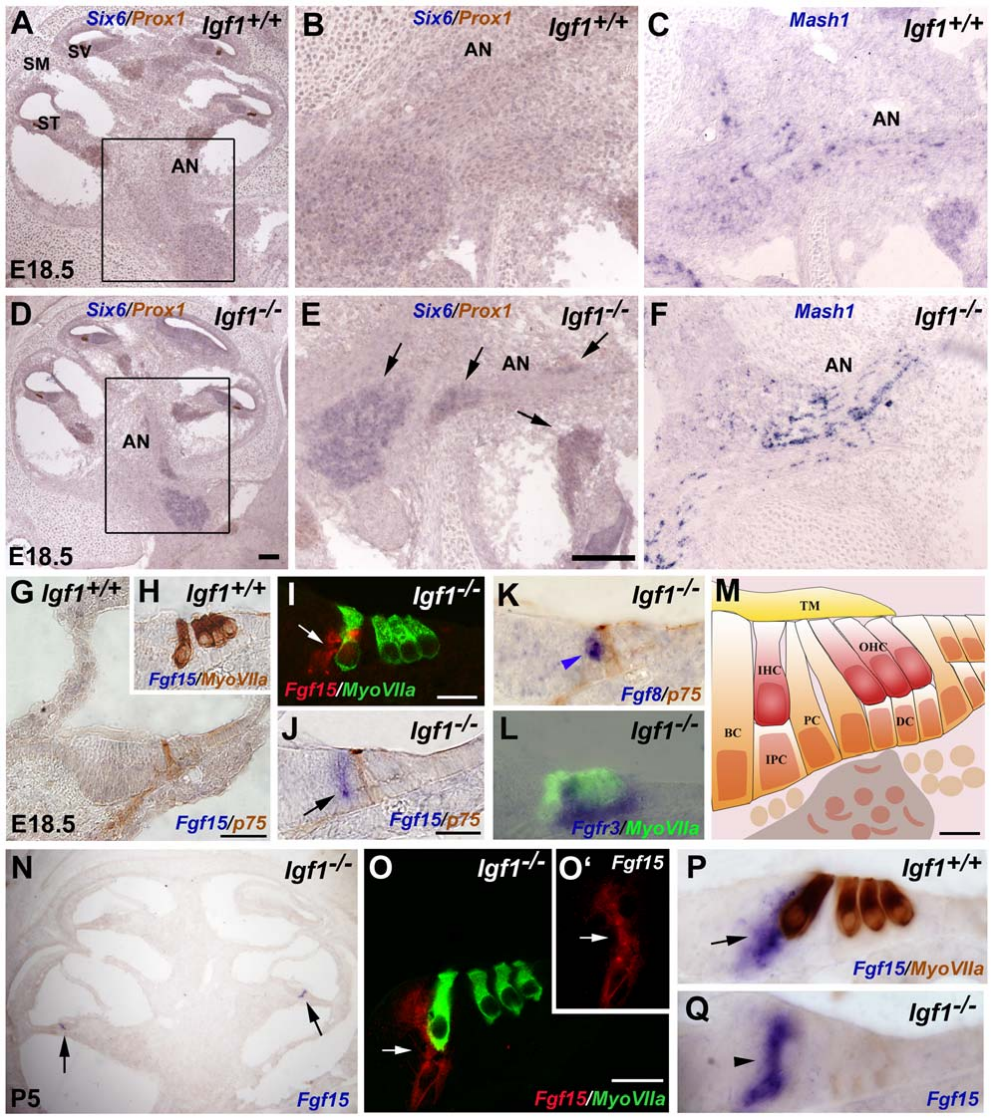
Up-regulation of *Six6*, *Mash1* and *Fgf15* in the embryonic cochlea of the *Igf1^−/−^* mouse. *In situ* hybridization for *Six6* (**A, B, D, E**), *Mash1* (**C, F**) *Fgf15* (**G–J and N–Q**), *Fgf8* (**K**) and *FgfR3* (**L**) transcripts was performed on cryostat sections from *Igf1^+/+^* and *Igf1^−/−^* E18.5 (A–F and G–M) and P5 (N–Q) cochleas. (**M**) Schematic drawing of the organ of Corti showing the different cell types at E18.5. *Six6* and *Mash1* expression was higher in the auditory nerve (AN) of E18.5 *Igf1^−/−^* cochlea. *Fgf15* mRNA expression located in the border cells (BC) and in the inner phalangeal cells (IPC) of E18.5 *Igf1^−/−^* mice (arrows in I,J), was absent in *Igf1^+/+^* mice (G,H). *Fgf8* (blue arrowhead in K) expression was detected in IHC in *Igf1^−/−^* and *FgfR3* (blue staining in L) transcripts were also detected in *Igf1^−/−^* supporting cells. At P5, *Fgf15* expression was observed in the IPC and BC in the basal turn of the cochlea of both *Igf1^−/−^* (arrows in N,O,O',P) and normal (arrowhead in Q) mice. Immunostaining for Prox1 (brown in A,B,D,E), MyosinVIIa (brown in H,P green in I,L,O) and p75 (brown in G,J,K) identified supporting cells, inner and outer hair cells and pillar cells respectively. Three embryos per genotype were tested in parallel in three independent experiments. DC, Deiter's cells; HC, hair cells; IHC, inner hair cells; OHC, outer hair cells; PC, pillar cells; SM, scala media; ST, scala tympani; SV, scala vestibuli; TM, tectorial membrane. Scale Bars: A, 100 µm (A,D,N); E, 100 µm (B, C, E, F); 50 µm (G); I, 20 µm (I,O,O'); and 30 µm (H,J,K,L,P,Q); M, 10 µm.

Parallel *in situ* hybridization studies confirmed the aberrant cellular expression of *Six6* and *Mash1*, and of the fibroblast growth factor *Fgf15*. These genes were expressed in the *Igf1^−/−^* cochlea despite being absent or expressed at very low levels in the *Igf1^+/+^* cochlea. Within the central nervous system, *Six6* is expressed in the presumptive and differentiating neural retina, ventral optic stalk, olfactory placodes, hypothalamus, and pituitary gland [36]. Although *Six6* mRNA is not normally expressed in the auditory nerve at E18.5 (Fig. 3A,B), transcripts were clearly detected in the *Igf1^−/−^* mouse (Fig. 3D,E, arrows in E). The proneural bHLH transcription factor *Mash1* was also more strongly expressed in the auditory nerve (AN) of the E18.5 *Igf1^−/−^* mouse (Fig. 3C,F), overlapping the glial transition zone [37]. At P5, *Mash1* expression was similar between genotypes and was associated with neuronal bodies amongst fibres that were strongly labelled for myelin basic protein, which may suggest that at this stage *Mash1* positive cells are root neurones [38] (Fig. S4). These results suggest that IGF-I participates in late neural cell fate decisions in the auditory ganglia.

Fibroblast growth factors and their receptors have key roles during inner ear development [39], [40]. At E15.5 and E18.5, *Fgf15* is not normally expressed in the organ of Corti of wild type mice but transcripts from this gene were found in the inner phalangeal cells and border cells of the *Igf1^−/−^* mice at E16.5 and E18.5, close to the inner hair cells in the basal turn of the cochlea (data not shown and Fig. 3G,J). These transcripts were associated with the specific markers p75 for pillar cells and myoVIIa for hair cells (Fig. 3I,J, see reference cartoon in M). At P5, *Fgf15* expression in the border and inner phalangeal cells was very similar between genotypes (Fig. 3N–Q). Expression of *Fgf8* (Fig. 3K) and *Fgfr3* (Fig. 3L) was unchanged in the *Igf1^−/−^* cochlea at E18.5.

**Figure 4:**
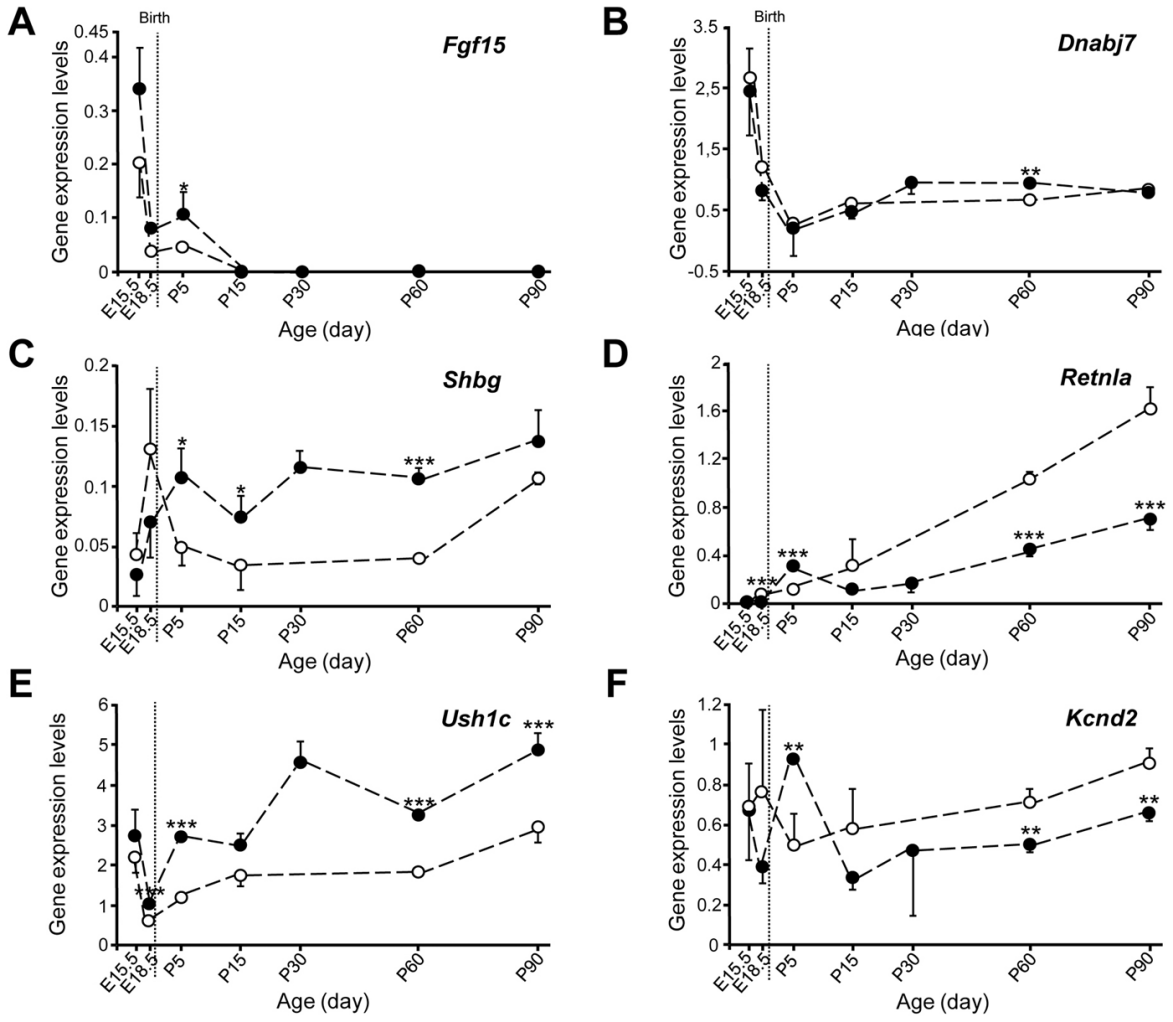
Time-course of IGF-I target gene mRNA expression. qRT-PCR analysis of mRNA from *Igf1^+/+^* (open circles) and *Igf1^−/−^* (closed circles) cochleae obtained at E15.5, E18.5, P5, P15, P30, P60 and P90. Eukaryotic 18S rRNA was used as the endogenous housekeeping control gene. Estimated gene expression levels are represented as 2^−ΔCt^·10^6^. *Fgf15* (**A**) and *Dnabj7* (**B**) expression profiles were similar, with high levels at E15.5 and lower levels from E18.5 onwards. Up to P5, more *Fgf15* transcripts and fewer *Dnabj7* transcripts were detected in the mutant cochlea. *Shbg* levels were higher from P5 onwards (**C**) whilst *Retnla* (**D**) expression increased with age. Although weaker in the *Igf1^−/−^* cochlea, *Ush1c* (**E**) mRNA levels were higher in the *Igf1^−/−^* cochlea and *Kcnd2* levels were lower from P15 onwards (**F**). Statistical significance estimated by Student's t-test was as follows ***p<0.005; **p<0.01; *p<0.05, with respect to wild type mice data (n = 6 mice/genotype).

The expression profiles of *Akr1c13*, *Dnabj7*, *Fgf15*, *Fibp*, *Foxg1*, *Foxm1*, *Kcnd2*, *Kif17*, *Mash1*, *Shbg*, *Retnla*, *Rp1h*, *Six6*, *Slc19a2* and *Ush1c* at stages E15.5, E18.5, P5, P15, P30, P60 and P90 were studied in the cochleas of *Igf1^+/+^* and *Igf1^−/−^* mice by qRT-PCR. They were categorised in three groups: i) genes with profiles that differed during embryonic development, either increasing like *Akr1c13*, *Fgf15*, *Foxm1* and *Six6* (Fig. 4A) or decreasing like *Dnabj7* (Fig. 4B), in the *Igf1^−/−^* cochlea; ii) genes with profiles that differed during the postnatal stages, increasing like *Fibp*, *Shbg* (Fig. 4C), *Slc19a2* and *Rp1h* or decreasing like *Kif17* and *Retnla* (Fig. 4D) in the *Igf1^−/−^* cochlea; iii) genes that were affected by the absence of *Igf1^−/−^* throughout embryonic and postnatal development, such as *Mash1*, *Ush1c* (Fig. 4E) and *Kcnd2* (Fig. 4F). One example from each category is shown (Fig. 4).

### FoxM1 and MEF2 Levels and Intracellular Localization Are Differentially Regulated in the *Igf1* ^−/−^ Mouse Cochlea

**Figure 5:**
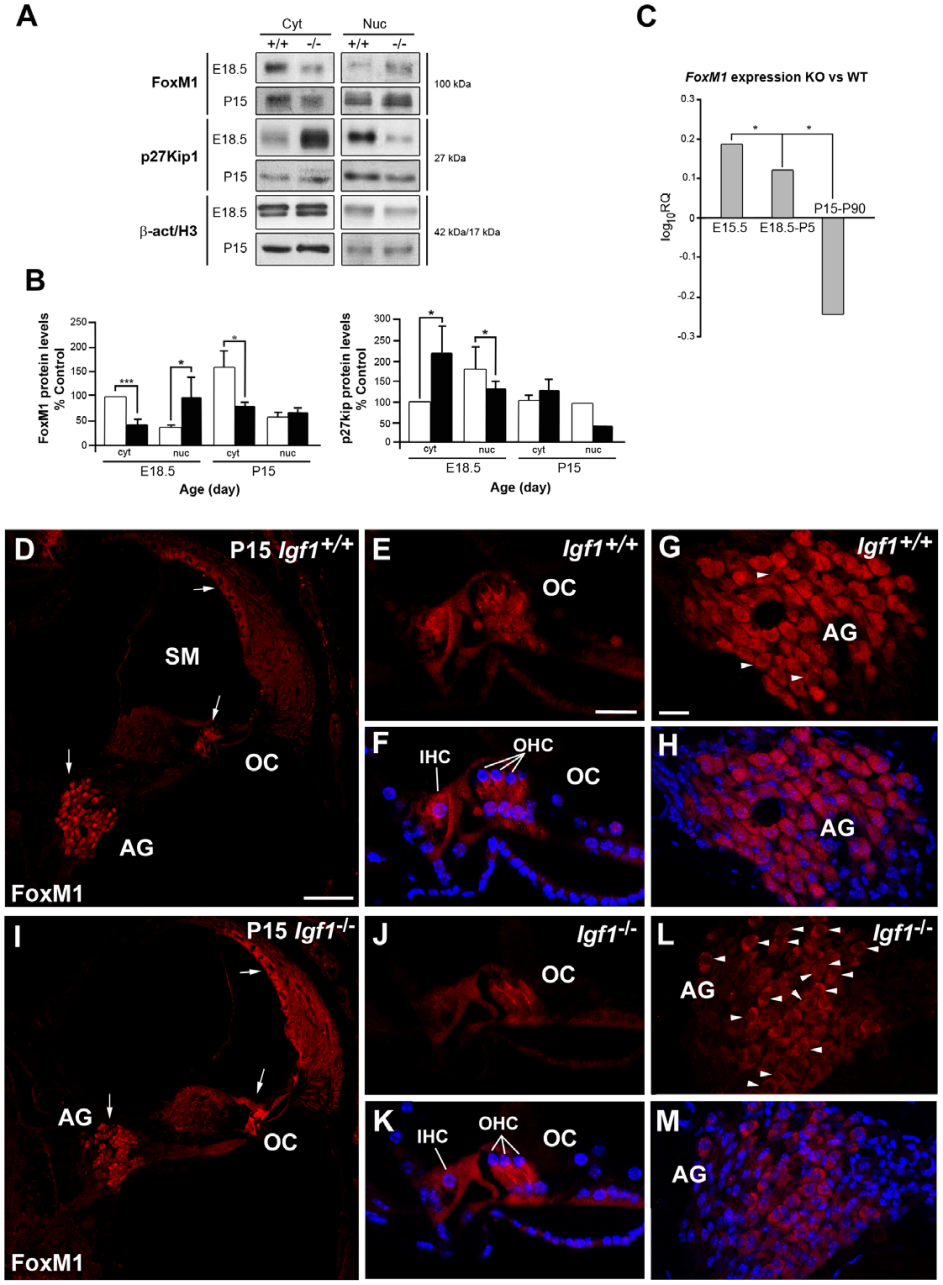
IGF-I deficiency modifies FoxM1 and p27^Kip1^ levels and intracellular localization. (**A**) Cytoplasmic and nuclear fractions of protein extracts obtained from at least 12 different E18.5 or P15 *Igf1^+/+^* or *Igf1^−/−^* mouse cochleas in at least six different experiments were immunoblotted to detect the presence of FoxM1 and p27^Kip1^. Blots were reprobed with β-actin (cytoplasmic fraction) or histone H3 (nucleus) as loading controls. The specific bands were measured by densitometry to determine the average expression with ImageJ software. Results were normalized by assigning a value of 100 to the cytoplasmic *Igf1^+/+^* and represented graphically in (**B**). (**C**) Relative quantification value (RQ) of *Foxm1* expression in the *Igf1^−/−^* cochlea compared to *Igf1^+/+^*, estimated by qRT-PCR at E15.5, E18.5-P5 and P15-P90. Data are presented as log_10_RQ average. (**D–M**) Localization of immunostaining for FoxM1 in the P15 *Igf1^+/+^* (D–H) and *Igf1^−/−^* (I–M) mouse cochlea. The expression was located in the AG, the stria vascularis and the organ of Corti (white arrows). Statistical significance estimated with the Student's t-test was: ***p<0.005; **p<0.01; *p<0.05, of mutant versus wild type mice data. Open and closed bars: *Igf1^+/+^* and *Igf1^−/−^* mice, respectively. Cyt, cytoplasm; Nuc, nucleus; AG, auditory ganglia, IHC, inner hair cell; OC, organ of Corti; OHC, outer hair cell; SM, scala media. Scale bars: D, 100 µm (D,I); E, 20 µm (E,F,J,K) and G, 20 µm (G, H, L, M).

In the mouse embryo, FoxM1 is associated with cell cycle control and DNA repair in neural progenitors and its expression decreases after differentiation [41]. FoxM1 prevents nuclear localization of the cyclin-dependent kinase inhibitor p27^Kip1^ and is essential for cytokinesis [42], [43]. In the E18.5 *Igf1* ^−/−^ mouse cochlea, *Foxm1* expression levels were up-regulated (+1.7 fold log_2_ change using Genechip Arrays and +1.2 with qRT-PCR) with respect to the wild type. To further explore the possible functional consequences of this altered *Foxm1* expression, cytoplasmic and nuclear protein extracts from the whole cochlea of E18.5 and P15 mice were analyzed. At E18.5, nuclear FoxM1 protein levels were 154% higher (p<0.05) in *Igf^−/−^* mice, whilst the cytoplasmic component was 57% lower (p<0.005, Fig. 5A,B). In contrast, at P15, FoxM1 protein levels were only 13% higher in the nuclear extract of whole cochlea, whilst the cytoplasmic fraction was 50% lower (p<0.05, Fig. 5B) Cytoplasmic protein levels for p27^Kip1^ were 121% higher (p<0.05) in *Igf^−/−^* mice at E18.5 whilst the nuclear fraction was 27% lower (p<0.05) (Fig. 5B). At P15, p27^Kip1^ cytoplasmic levels were 24% higher in the *Igf1* ^−/−^ mouse and the nuclear fraction was 58% lower. These differences in subcellular localization of both FoxM1 and p27^Kip1^ in the *Igf1* ^−/−^ mouse cochlea suggested that FoxM1 may modulate the nuclear targeting of p27^Kip1^. The relative expression levels of *FoxM1* remained high during development and decreased at postnatal stages in the *Igf1* ^−/−^ with respect to the *Igf1* ^+/+^cochlea (Fig. 5C). At P15, FoxM1 protein was located in both genotypes at the stria vascularis, the auditory ganglia and in the organ of Corti (Fig. 5D–M).

**Figure 6:**
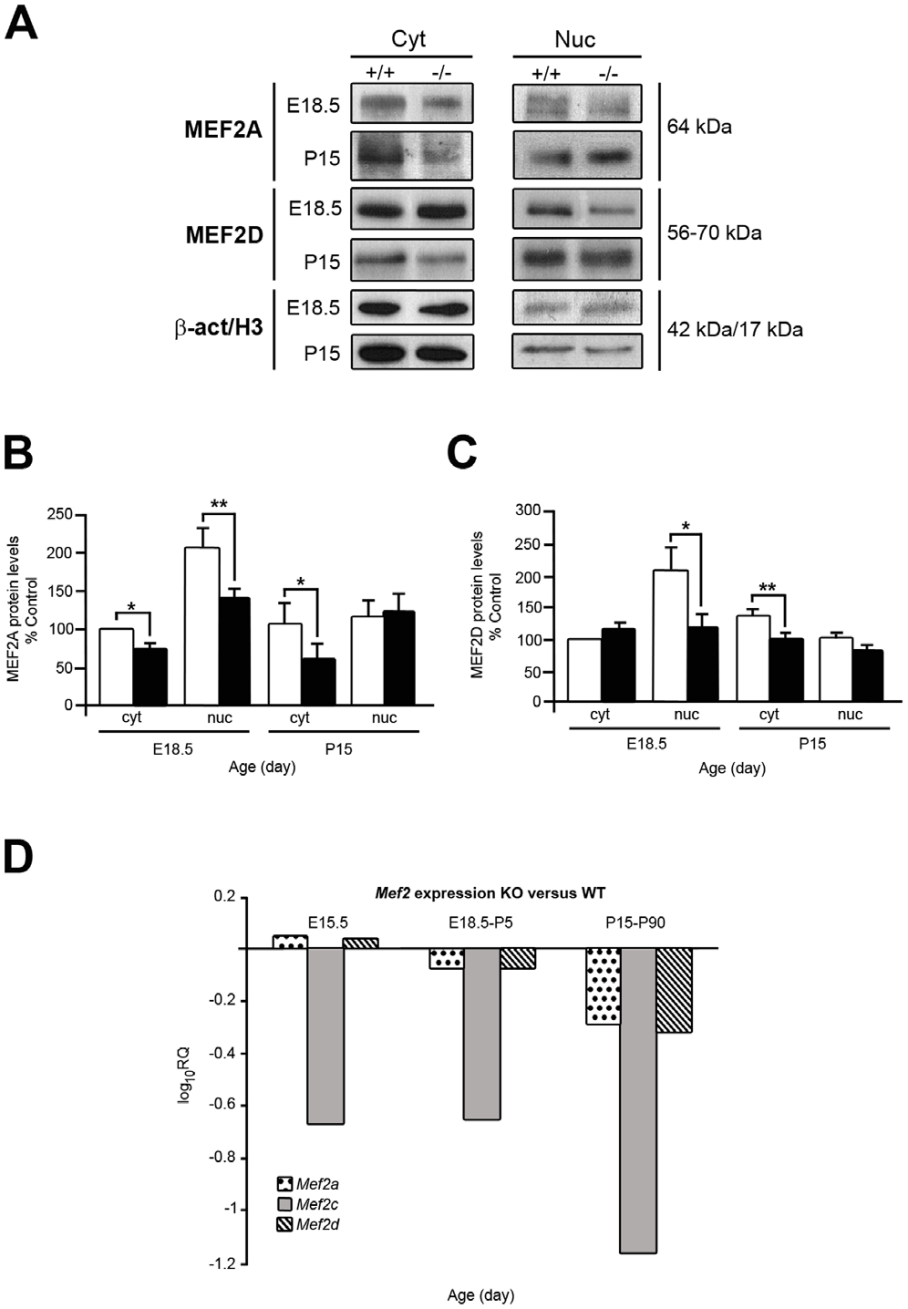
IGF-I deficiency modifies MEF2 levels and intracellular localization. (**A**) Cytoplasmic and nuclear fractions of protein extracts obtained from E18.5 and P15 normal or *Igf1^−/−^* mouse cochleae (n = 21, from at least 7 different experiments) were immunoblotted to detect the presence of MEF2A and MEF2D. Blots were re-probed with β-Actin (cytoplasmic fraction) or histone H3 (nucleus) as loading controls. The specific bands were measured by densitometry (ImageJ software) to determine the average expression. Results were normalized respect to β-actin or histone, a value of 100 was assigned to the scanned intensity of cytoplasmic forms in *Igf1^+/+^* and represented graphically in (**B,C**). (**D**) *Mef2a*, *Mef2c* and *Mef2d* expression was measured by qRT-PCR at E15.5, E18.5-P5 and P15-P90 data points in the *Igf1^−/−^* mouse cochleas and compared with the *Igf1^+/+^*. Data are presented as the mean of log_10_RQ. Statistical significance estimated with Student's t-test was: ***p<0.005; **p<0.01; *p<0.05. Open bars: *Igf1^+/+^* mouse; Closed bars: *Igf1^−/−^* mouse. Cyt, cytoplasm; Nuc, nucleus, β-act, β-actine; H3, histone 3.

**Figure 7:**
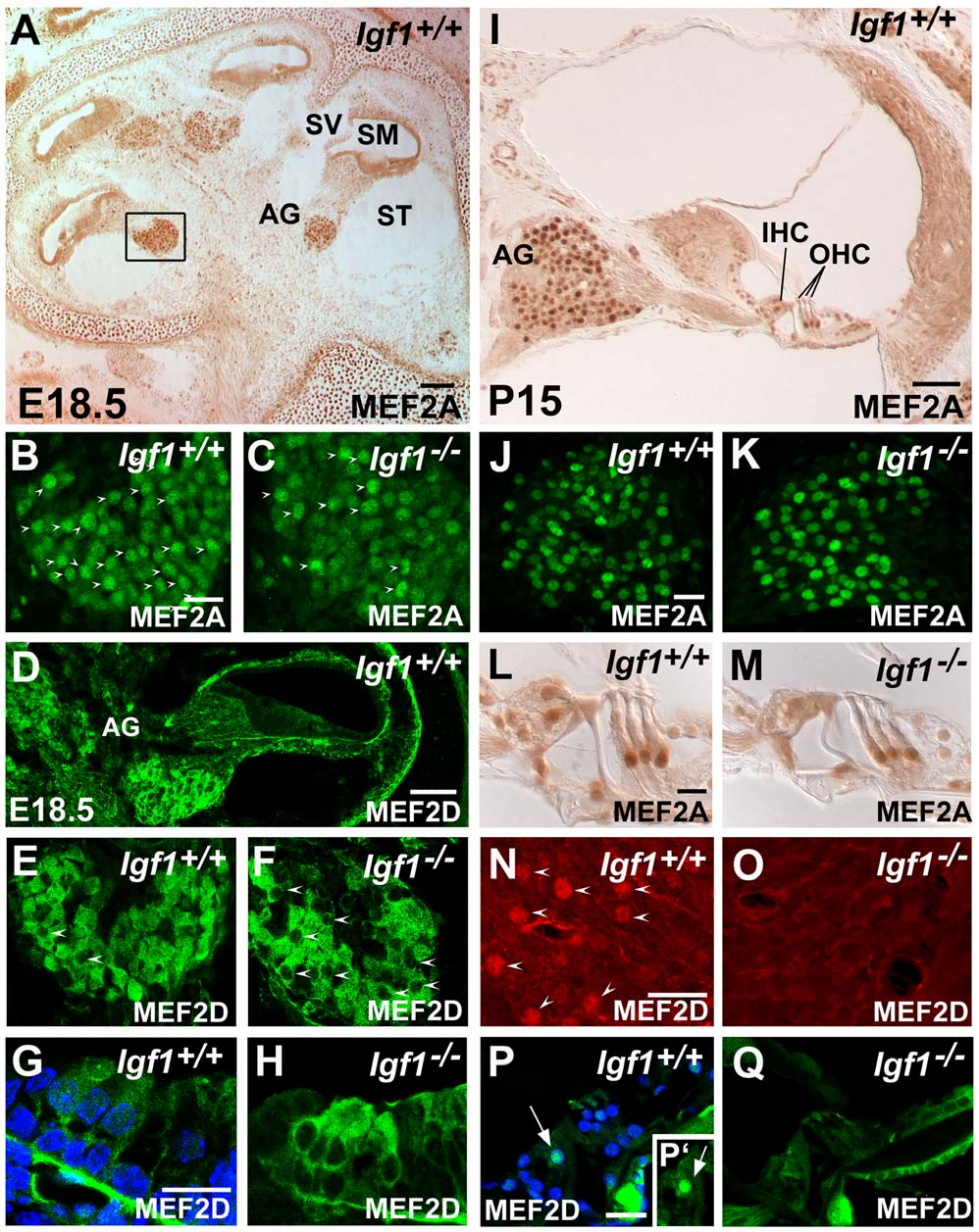
MEF2A and MEF2D immunolocalization in the cochlea of *Igf1^+/+^* and *Igf1^−/−^* mice. MEF2A expression in the cochlea of E18.5 (A,B,C) and P15 (I,J,K,L,M) *Igf1^+/+^* (A,B,I,J) and *Igf1^−/−^* (C,K) mice. At E18.5, MEF2A strongly stained the nuclei in the *Igf1^+/+^* auditory ganglion (arrowheads in B), whereas fewer nuclei appeared labelled in the *Igf1^−/−^* ganglia (arrowheads in C) where the staining appeared more cytoplasmatic. At P15, labelling was similar in the neurones (J,K), Deiter's cells, pillar cells and in the IHC (L,M) of both genotypes. MEF2D expression at E18.5 was shown in the auditory ganglia (E,F) and organ of Corti (G,H). MEF2D expression was less nuclear in the *Igf1^−/−^* (F, arrowheads pointing to unlabelled nuclei, H) than in the wild type mouse (E,G). At P15, MEF2D expression was observed in the nuclei of auditory neurons (N) and in the IHC (P,P') in the *Igf1^+/+^* but not in the *Igf1^−/−^* mouse (O,Q). AG, auditory ganglion; IHC, inner hair cells; OHC, outer hair cells; SM, scale media; ST, scala tympani; SV, scala vestibule. Scale bars:. A, 100 µm; B, 25 µm (B,C,E,F); D, 75 µm; I, 100 µm; G, 20 µm (G,H); J, 20 µm (J,K); L, 20 µm (L,M); N, 20 µm (N,O); P, 20 µm (P,P',Q).

To further identify common transcriptional regulators of the IGF-I cochlear target genes, the 5′UTR promoter regions (1.5 kb upstream) of the selected genes were searched using MEME software. Those with the lowest p-value for random recurrence were selected and were analysed with TESS. Finally, the consensus sequences with the highest probability of matching motifs were selected. From two different analyses of genes up-regulated in the *Igf1^−/−^* cochlea a possible binding site for the MEF2 transcription factor was identified with the strongest likelihood of alignment. There are four members of the MEF2 family of transcription factors, A to D, whose tissue expression and functions are not well known [44]. MEF2A and D are expressed in neuronal progenitors and specific neuronal populations and *Mef2c^−/−^* mice show aberrant neuronal migration during development and immature adult neurons [45]. In muscle cells, MEF2 levels are modulated by IGF-I, which delays MEF2 degradation by the ubiquitin-dependent proteasome pathway and promotes MEF2 translocation to the nucleus [46]. Here we study the cochlear expression and regulation of MEF2 family members by IGF-I (Fig. 6). Western blot studies showed that the levels and subcellular location of MEF2 were altered in the *Igf1^−/−^* cochlea when compared to the wild type control (Fig. 6A,B,C). At E18.5, MEF2A protein levels were 26% lower in the cytoplasm (p<0.05) and 32% lower (p<0.01) in the nucleus, whereas, at P15 both cytoplasmic and nuclear levels were 40% lower (p<0.05) (Fig. 6B). On the other hand, MEF2D protein levels in the E18.5 *Igf1^−/−^* cochlea were similar in the cytoplasm but 43% lower (p<0.05) in the nucleus, while at P15, they were lower in both fractions (25%, p<0.01, and 19% in the cytoplasm and nuclear fractions, respectively) (Fig. 6C). *Mef2a*, *Mef2c* and *Mef2d* temporal expression profiles were studied in the cochlea of *Igf1^+/+^* and *Igf1^−/−^* mice by qRT-PCR from E15.5 to P90 (Fig. 6D). *Mef2a* and *Mef2d* showed similar differences in expression level in embryonic and adult tissue, being slightly higher in *Igf1^−/−^* cochleae at E15.5 and lower at P5 and P90. In contrast, *Mef2c* expression levels were much lower at all ages and MEF2C protein was undetectable. Fig. 7 shows the localization of MEF2A and D immunostaining in the E18.5 and P15 mouse cochlea of both genotypes. MEF2A immunostaining was strong in E18.5 auditory ganglion neurons, although it presented a more diffuse cellular pattern in immature neurons of *Igf1* ^−/−^ mice (Fig. 7A,B,C). At P15, MEF2A immunostaining was visible in the auditory ganglion (Fig. 7I,J,K), pillar cells, Deiter's cells and inner hair cells (Fig. 7L,M) of both genotypes. MEF2D expression was observed in the auditory ganglia and organ of Corti at E18.5 (Fig. 7D,E,F,G,H) and P15 (Fig. 7N,O,P,Q). At E18.5, MEF2D label was strong and mostly located in the cytoplasm of the hair cells in the *Igf1* ^−/−^ (Fig. 7H), whereas at P15 immunostaining decreased in the null mouse (Fig. 7O,Q) and was strongly nuclear in the neurons and inner hair cells of the wild type cochlea (Fig. 7N,P).

## Discussion

We have studied the molecular mechanisms by which IGF-I regulates cochlear development and maturation by analyzing the following parameters in the *Igf1^+/+^* wild type and *Igf1^−/−^* null mouse cochleae: i) the spatiotemporal expression of IGF-system factors, receptors and binding proteins; ii) the activation of the main IGF-I signalling kinases Akt, ERK and p38; iii) total cochlear transcriptome changes caused by IGF-I-deficit by using mRNA arrays; and iv) transcription factors associated with neuronal cell cycle regulation modulated by IGF-I availability. We have found novel regulatory genes for cochlear development whose normal expression and activation depends on IGF-I. Severe syndromic deafness in man is associated with null mutations in *IGF1* [9], [10], [11] and also with low levels of IGF-I [47]. Accordingly, the *Igf1* ^−/−^ mouse shows poor growth rates, high mortality, profound sensorineural deafness and late postnatal morphological alterations in the cochlea [16] We have shown previously that the absence of IGF-I causes poor myelination and delayed maturation of auditory neurones that suffer apoptosis during the early postnatal mouse development P5-P20 [17], [18]. At birth, however, the *Igf1* ^−/−^ mouse cochlea is the normal size with the expected complement of cell types in the organ of Corti. At the molecular level, signs of delayed differentiation were obvious, but the molecular clues underlying this cochlear-specific phenotype were not clear. Here we show that IGF-I deficit could be compensated, at least in part, by increased expression of its high affinity receptor, which can also be activated by other insulin family factors, whose gene expression levels were unchanged. Typical IGF-I intracellular target kinases were also examined in the cochlea, and interestingly a 25% reduction in the activated forms of prosurvival Akt kinase and proliferation-associated ERK1/2 were found, with a dramatic increase in the levels of the stress kinase p38. Further analysis to uncover IGF-I targets in the cochlea was carried out by using gene microarrays to do a comparative analysis of the expression profiles of the developing cochlea in *Igf1^+/+^* and *Igf1^−/−^* mice.

**Figure 8:**
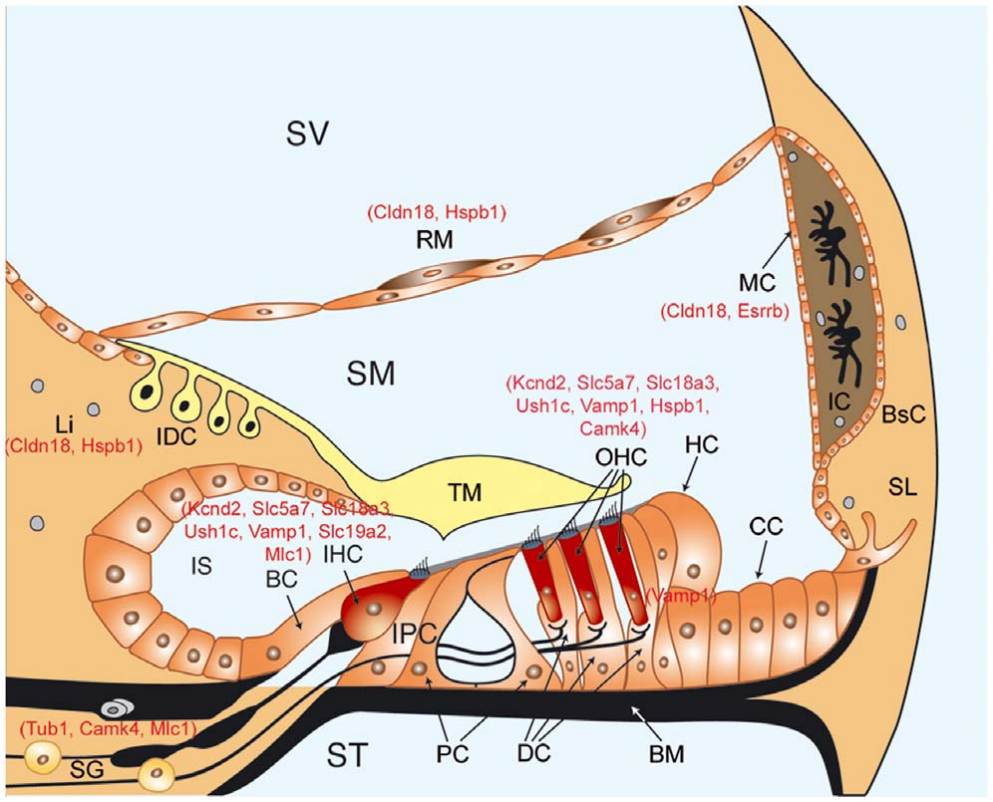
Differentially expressed genes in the IGF-I-deficient cochlea. Names of selected differentially expressed genes (red) are shown on a schematic drawing of the adult scala media. BC, border cells; BsC, basal cells; BM, basilar membrane; CC, Claudius's cells; DC, Deiter's cells; HC, Hensen's cells; IC, intermediate cells; IDC, interdental cells; IHC, inner hair cells; IPC, inner phalangeal cells; IS, inner sulcus; Li, spiral limbus; MC, marginal cells; OHC, outer hair cells; PC, pillar cells; RM, Reisner's membrane; AG, auditory ganglion; SL, spiral ligament; SM, scala media; ST, scala tympani; SV, scala vestibuli; TM, tectorial membrane.

Here, we have identified 231 genes that are differentially expressed in the cochlea of the *Igf1* ^−/−^ mouse. A subset of these genes was further studied by using a combination of complementary approaches to further understand IGF-I actions in the inner ear. To our knowledge, this is the first time that a comparative gene expression profile has been carried out in an *Igf1* ^−/−^ mouse tissue. Fig. 8 schematically shows the localization of the differentially expressed genes in *Igf1* ^−/−^ cochleas that are known to be important for inner ear development or to be linked to inherited deafness (9% of total), including *Kcnd2*, *Slc19a2* and *Ush1c*. The later encodes the stereociliary protein harmonin and mutations in this gene cause Usher's Syndrome 1C [32]. Interestingly, the syndrome includes retinal degeneration, which is also associated with mutations in *Rp1h* [34], a gene expressed at higher levels in the *Igf1^−/−^* cochlea. Over-expression of IGF-I causes profound alterations in the vascularisation of the mouse eye [48] but to our knowledge there are no reports of eye defects associated with IGF-I deficiency. In contrast, IGF-I deficit in the mouse severely impairs normal development of the olfactory bulb [49]. 91% of the genes we found differentially expressed in the *Igf1^−/−^* cochlea had not been described previously in the inner ear. For example, *Fgf15*, the ortholog of human and chicken *Fgf19*, presented an expression pattern suggestive of a novel contribution to cell fate specification within the sensory epithelia. This raises the question of the specific role of this member of the FGF family during inner ear development and of its possible regulation by IGF-I [12].

Several transporters fundamental for the traffic of synaptic vesicles were also expressed differentially in the *Igf1^−/−^* cochlea, which is consistent with previous observations of aberrant synapsis at the inner hair cells [17]. For example, the thiamine transporter, *Slc19a2* [32], [50], the choline and acetylcholine transporters, *Slc5a7* and *Slc18a3* [51], and the membrane protein *Vamp1* were normally expressed in the inner hair cells of the mouse but their levels were consistently lower in the *Igf1* ^−/−^ cochlea. In contrast *Mlc1*, which encodes a protein located in the afferent fibers of the inner hair cells [52], was expressed at higher levels. These data support the idea that IGF-I is a key molecule for the maturation of the auditory neurons and the refinement of the synaptic connections at the inner hair cells.

Alterations in ion homeostasis and transport were also associated with IGF-I deficit because *Kcnd2*, *Kif17*, *Kcnmb1* and *Cacna1f* showed lower levels in *Igf1^−/−^* cochleae. *Kcnd2* encodes the K+ channel Kv4.2, which is expressed in neurons that innervate apical hair cells, which regulates dendritic excitability [53] and which is transported to the dendrites by the neuronal kinesin *Kif17* [54]. The calcium-activated potassium channel *Kcnmb1* is know to be expressed in the cochlea but the null mouse has no obvious cochlear phenotype or hearing impairment [55]. In contrast, the presence of the calcium channel *Cacna1f* had not been described previously in the mouse cochlea, but mutations in man and mouse cause retinal neurotransmission disorders [56], [57]. In addition, differentially regulated genes included Claudin 18 a tight junction protein expressed in the stria vascularis [2] and the estrogen related receptor Esrrb, whose mutations in man cause autosomal-recessive non syndromic hearing impairment and that is expressed and controls the development of the strial marginal cells [20], [21]. These data taken together suggest that ion homeostasis and vesicular transport are impaired in the deaf *Igf1^−/−^* mouse.

Neuronal fate specification is mastered by transcription factors like *Six6* and *Mash1*, which are typically expressed in the central nervous system. *Six6* is a member of the Six/sine oculis family and it is known to be expressed in the developing and adult retina, in the optic nerve, and in the hypothalamic and pituitary regions [58], [59]. *Mash1* is a proneural transcription factor of the basic helix-loop-helix family, which participates in the commitment of neural progenitors, promotion of cell cycle exit and neuronal migration, and in the final specification of neuronal identities in the brain [60], [61]. Interestingly, both were increased in the *Igf1^−/−^* mouse embryonic cochlea, *Mash1* transcripts were visible in the central part of the auditory nerve at the glial transition zone [37], where Atoh1, another member of the bHLH transcription factor family [62], has been shown to play a central role in root neurons survival and on the functional maintenance of the peripheral and central auditory pathway [63]. Taken together these data suggest that these bHLH transcription factors are key players for the differentiation and survival of the neurons at the interface between the peripheral and central nervous systems.

IGF-I promotes a faster transition of the otic neural progenitors to a mature neuronal state in the developing chicken inner ear [12], [64]. These data taken together suggest that IGF-I represses the expression of *Six6* and *Mash1* during normal inner ear development either directly or indirectly to facilitate neuronal differentiation.

It is known that IGF-I is a key factor for cell cycle progression and DNA repair and several cell types in the *Igf1^−/−^* mouse, including cochlear neurones, are smaller and more immature than those of the wild type mouse [17], [18], [65]. Here we show that in the cochlea, IGF-I deficit causes an increase in IGF1R expression levels although there is a net reduction in the ratio of tyrosine phosphorylation, an increase in the activated phospho-p38 stress kinase, and a decrease in the levels of the active phosphorylated forms of the kinases ERK1/2 and Akt, the main intracellular executors of IGF-I actions, indicating that the balance between cell proliferation, survival and differentiation is altered. However, the complexity of the regulation of cellular processes was evidenced by the contrasting increase in the expression levels of the forkhead transcription factor *Foxm1*. FoxM1 is essential for mitotic progression and for the transcriptional response during DNA damage/checkpoint signalling [42], [66], [67]. Its presence in the developing cochlea had not been reported previously. Its increased activation in the *Igf1^−/−^* cochlea was confirmed by its nuclear localization and by the inhibition of one of its downstream targets, the cyclin-dependent kinase inhibitor p27^Kip1^. In contrast, transcripts for other cell cycle proteins, such as INCENP, were expressed at lower levels, suggesting problems in chromosome segregation [68]. In early postnatal cochleae from *Igf1^−/−^* mice there is increased neuronal apoptosis and delayed neuronal maturation, but there is no evidence for altered cellular proliferation or cell damage [17]. These data suggest that FoxM1 activation compensates for the unbalanced progression through the cell cycle caused by IGF-I deficit.

Further insight into IGF-I cochlear targets was obtained by *in silico* analysis of the promoters of IGF-I-modulated genes that unveiled a potential role for MEF2 and its modulation by IGF-I in the cochlea. MEF2 is essential for myogenic and neuronal differentiation [45], [69]. In both cell types, IGF-I activates MEF2 by decreasing its degradation rate and by preventing its translocation from the nuclei to the cytoplasm [46], [69]. MEF2D is expressed in sensory neurons during development and it is regulated by a TrkA-dependent ERK5 pathway that promotes neuronal survival [70]. MEF2 is activated by the Raf/MAPK cascade [46] and also by p38 MAPK [71]. Here we show that MEF2A and D but not C are highly expressed in the nuclei of embryonic mouse auditory ganglion neurons and that nuclear MEF2 protein levels are lower in the developing *Igf1* ^−/−^ cochlea. These data reinforce the conclusion that MEF2A and D are key targets for otic IGF-I action and that they may have a fundamental role during cochlear development. To our knowledge, this is the first time that MEF2 transcription factors expression has been reported in the auditory ganglia.

In summary, we show that *Igf1* and *Igf1r* are expressed in the developing mouse cochlea with complementary cellular patterns. The stria vascularis apparently provides an intra-cochlear source of IGF-I. Analysis of IGF-I-deficient cochlea showed that the signalling levels of Akt and ERK1/2 were lower and that p38 activation was significantly higher. Transcriptional profiling of the *Igf1* ^−/−^ cochlea identified potential novel IGF-I targets, including factors like *Six6*, *Mash1* and *Fgf15*. Finally, the transcription factors *FoxM1*, *Mef2a* and *Mef2d* are expressed in the developing inner ear and their sub-cellular localisation is modulated by IGF-I availability. The results presented here offer new insight into the mechanisms by which IGF-I support sensory cell and neuronal survival and differentiation in the auditory receptor, and reveal novel regulatory mechanisms of the cell cycle during cochlear development.

## Supporting Information

Figure S1 Spatiotemporal expression patterns of *Igf1r* mRNA and IGF1R protein in the *Igf1^+/+^*and *Igf1^−/−^* mouse cochlea. (A–F) shows that the mRNA expression of *Igf1r* was identical in the two genotypes. (A,B,C) P5 *Igf1^+/+^* and (D,E,F) P5 *Igf1^−/−^* mice. IGF1R protein was shown at the organ of Corti and in the auditory ganglion at E15.5 (G,H) and E18.5 (I,I',J,J') with similar cellular localization between genotypes. At P15, the expression was located mainly in the neurons of the auditory ganglion (K,L), no differences could be observed. AG, auditory ganglion; BsC, basal cells IHC, inner hair cells; MC, marginal cells; OC, organ of Corti; OHC, outer hair cells; SM, scale media; ST, scala tympani; SV, scala vestibule, TM, tectorial membrane. Scale bars: A, 150 µm (A,D); B, 20 µm (B,C,E,F); G, 20 µm (G,H); I, 100 µm (I,J); I', 20 µm (I',J'); K, 20 µm (K,L).
(3.53 MB DOC)

Figure S2 IGF-I deficiency modifies IGF1R phosphorylation levels.Protein extracts obtained from E15.5, E18.5, P5, P60 and P90 *Igf1^+/+^* or *Igf1^−/−^* mouse cochleas (n = 6, from at least 2 different experiments) were immunoblotted to detect the presence of pIGF1R. Blots were re-probed with IGF1R as loading control. The specific bands were measured by densitometry (ImageJ software) to determine the average expression. Results were normalized, a value of 100 was assigned to the scanned intensity of the E15.5 *Igf1^+/+^* cochlear extract. Statistical significance estimated with Student's t-test was: **p<0.01; *p<0.05. Open bars: *Igf1^+/+^* mouse; Closed bars: *Igf1^−/−^* mouse.
(0.05 MB TIF)

Figure S3 Clustering of the differentially expressed genes in *Igf1^−/−^* cochleas grouped according to their functional category. The 231 genes differentially expressed (closed bars), were classified by their biological annotation compared with all *Mus musculus* genome annotations in the NCBI (open bars). The statistical analysis of the biological processes included a Bonferroni correction for multiple testing and processes were selected at p<0.05. The differentially expressed genes are implicated in the following biological processes: signal transduction, developmental process, ligand-mediated signalling, cell communication, immunity and defence, cytokine and chemokine mediated signalling, muscle contraction, lipid, fatty acid and steroid metabolism, granulocyte-mediated immunity and homeostasis.
(0.20 MB TIF)

Figure S4 *Mash1* expression in the *Igf1^+/+^*and *Igf1^−/−^* P5 mouse auditory nerve.(A–K) *Mash1* in situ hybridization was performed on cryostat sections in *Igf1^+/+^* (A–G) and *Igf1^−/−^* (H–K) cochleas at P5. Axons in the auditory nerve (AN) were recognised by 3A10 immunostaining (red C,D,F,G,J,K). At P5, *Mash1* expression in the auditory nerve did not show differences between genotypes (B–G,I–K). B, E and I are higher magnification images of the boxed areas in A and H, respectively. D,G,K are merge images of *Mash1* and 3A10 labelling. (L–S) Double in situ hybridization of *Mash1* (red; L,P,O,S) and inmunohistochemistry of myelin basic protein (green, MBP; M,Q,O,S). Cell nuclei were stained with DAPI (blue; N,R,O,S). *Mash1* expression was perinuclear and was associated at P5 to the soma of cells, probably root neurones, embedded in auditory axons (white arrows; L–S). Three embryos per genotype were tested in parallel in three independent experiments. AN, auditory nerve; SM, scala media; ST, scala tympani; SV, scala vestibuli. Scale bars: H, 150 µm (A, H); K, 30 µm (B–G,I–K), L, 35 µm (L–O); P, 20 µm (P–S).
(7.24 MB TIF)

Table S1 Summary of inventoried TaqMan probes used for qRT-PCR. Applied Biosystems: https://products.appliedbiosystems.com/ab/en/US/adirect/ab?cmd=ABGEKeywordSearch.
(0.06 MB DOC)

Table S2 Summary of cDNAS used to generate the *in situ* hybridization probes. Prior to the *in situ* hybridization, all clones were sequenced (ABI 3130XL Applied Biosystems). At least 3 embryos per genotype were tested in parallel in three independent experiments. No signal was obtained with the sense probe (data not show) * *Igf1r* probe was the generous gift of Prof. Flora de Pablo (CIB, CSIC, Madrid) ^1^References 1. Lopez-Rios J, Gallardo ME, Rodriguez de Cordoba S, Bovolenta P (1999) Six9 (Optx2), a new member of the six gene family of transcription factors, is expressed at early stages of vertebrate ocular and pituitary development. Mech Dev 83: 155–159. 2. Guillemot F, Joyner AL (1993) Dynamic expression of the murine Achaete-Scute homologue Mash-1 in the developing nervous system. Mech Dev 42: 171–185. 3. McWhirter JR, Goulding M, Weiner JA, Chun J, Murre C (1997) A novel fibroblast growth factor gene expressed in the developing nervous system is a downstream target of the chimeric homeodomain oncoprotein E2A-Pbx1. Development 124: 3221–3232. 4. Hayashi T, Cunningham D, Bermingham-McDonogh O (2007) Loss of Fgfr3 leads to excess hair cell development in the mouse organ of Corti. Dev Dyn 236: 525–533. 5. Ladher RK, Wright TJ, Moon AM, Mansour SL, Schoenwolf GC (2005) FGF8 initiates inner ear induction in chick and mouse. Genes Dev 19: 603–613. 6. Liu JP, Baker J, Perkins AS, Robertson EJ, Efstratiadis A (1993) Mice carrying null mutations of the genes encoding insulin-like growth factor I (Igf-1) and type 1 IGF receptor (Igf1r). Cell 75: 59–72.
(0.05 MB DOC)

Table S3 Summary of antibodies used for immunohistochemistry and Western blotting. For IHC, at least 3 embryos per genotype were tested in parallel in three independent experiments. Control experiments without primary antibody were carried out for each reaction and indicated that the staining pattern was specific for antigen recognition (data not shown). ^1^Antibody type: RbP, rabbit polyclonal; MouM mouse monoclonal; GP, goat polyclonal Abbreviations: DSHB, Developmental Studies Hybridoma Bank; IHC, immunohistochemistry; WB, western blotting.
(0.06 MB DOC)

Table S4 Differentially expressed genes in the E18.5 cochlea of the *Igf1^−/−^* mutant mouse ranked by gene ontology and biological process annotations. Genes were selected according to reported inner ear expression, their relation with deafness, biological function determined with the PANTHER and FATIGO programmes, as well as fold-change and low-variance calculated with multi-mgMOS software. nf, not described previously in the literature; nd, not determined by real-time PCR ^1^Average fold change from microarray experiments. ^2^Fold change determined by real-time PCR. nd, not determined; nv, not verified ^3^References 1. Coppens AG, Kiss R, Heizmann CW, Schafer BW, Poncelet L (2001) Immunolocalization of the calcium binding S100A1, S100A5 and S100A6 proteins in the dog cochlea during postnatal development. Brain Res Dev Brain Res 126: 191–199. 2. Kitajiri SI, Furuse M, Morita K, Saishin-Kiuchi Y, Kido H, et al. (2004) Expression patterns of claudins, tight junction adhesion molecules, in the inner ear. Hear Res 187: 25–34. 3. Fritzsch B, Pauley S, Beisel KW (2006) Cells, molecules and morphogenesis: the making of the vertebrate ear. Brain Res 1091: 151–171. 4. Chu PJ, Rivera JF, Arnold DB (2006) A role for Kif17 in transport of Kv4.2. J Biol Chem 281: 365–373. 5. Sokolowski BH, Sakai Y, Harvey MC, Duzhyy DE (2004) Identification and localization of an arachidonic acid-sensitive potassium channel in the cochlea. J Neurosci 24: 6265–6276. 6. Adamson CL, Reid MA, Mo ZL, Bowne-English J, Davis RL (2002) Firing features and potassium channel content of murine spiral ganglion neurons vary with cochlear location. J Comp Neurol 447: 331–350. 7. Langer P, Grunder S, Rusch A (2003) Expression of Ca2+-activated BK channel mRNA and its splice variants in the rat cochlea. J Comp Neurol 455: 198–209. 8. Ruttiger L, Sausbier M, Zimmermann U, Winter H, Braig C, et al. (2004) Deletion of the Ca2+-activated potassium (BK) alpha-subunit but not the BKbeta1-subunit leads to progressive hearing loss. Proc Natl Acad Sci U S A 101: 12922–12927. 9. Teijido O, Casaroli-Marano R, Kharkovets T, Aguado F, Zorzano A, et al. (2007) Expression patterns of MLC1 protein in the central and peripheral nervous systems. Neurobiol Dis 26: 532–545. 10. Previati M, Lanzoni I, Corbacella E, Magosso S, Giuffre S, et al. (2004) RNA expression induced by cisplatin in an organ of Corti-derived immortalized cell line. Hear Res 196: 8–18. 11. Bergeron AL, Schrader A, Yang D, Osman AA, Simmons DD (2005) The final stage of cholinergic differentiation occurs below inner hair cells during development of the rodent cochlea. J Assoc Res Otolaryngol 6: 401–415. 12. Ladher RK, Anakwe KU, Gurney AL, Schoenwolf GC, Francis-West PH (2000) Identification of synergistic signals initiating inner ear development. Science 290: 1965–1967. 13. Sanchez-Calderon H, Francisco-Morcillo J, Martin-Partido G, Hidalgo-Sanchez M (2007) Fgf19 expression patterns in the developing chick inner ear. Gene Expr Patterns 7: 30–38. 14. Zheng W, Huang L, Wei ZB, Silvius D, Tang B, et al. (2003) The role of Six1 in mammalian auditory system development. Development 130: 3989–4000. 15. Carroll K, Gomez C, Shapiro L (2004) Tubby proteins: the plot thickens. Nat Rev Mol Cell Biol 5: 55–63. 16. Boeda B, El-Amraoui A, Bahloul A, Goodyear R, Daviet L, et al. (2002) Myosin VIIa, harmonin and cadherin 23, three Usher I gene products that cooperate to shape the sensory hair cell bundle. Embo J 21: 6689–6699. 17. Safieddine S, Wenthold RJ (1999) SNARE complex at the ribbon synapses of cochlear hair cells: analysis of synaptic vesicle- and synaptic membrane-associated proteins. Eur J Neurosci 11: 803–812. 18. Liberman MC, Tartaglini E, Fleming JC, Neufeld EJ (2006) Deletion of SLC19A2, the high affinity thiamine transporter, causes selective inner hair cell loss and an auditory neuropathy phenotype. J Assoc Res Otolaryngol 7: 211–217. 19. Gong T-WL, I. Jill Karolyi, James MacDonald, Lisa Beyer, Yehoash Raphael, et al. (2006) Age-Related Changes in Cochlear Gene Expression In Normal and Shaker 2 Mice JARO 7: 317–328. 20. Chen J, Nathans J (2007) Estrogen-related receptor beta/NR3B2 controls epithelial cell fate and endolymph production by the stria vascularis. Dev Cell 13: 325–337. 21. Collin RW, Kalay E, Tariq M, Peters T, van der Zwaag B, et al. (2008) Mutations of ESRRB encoding estrogen-related receptor beta cause autosomal-recessive nonsyndromic hearing impairment DFNB35. Am J Hum Genet 82: 125–138. 22. Stelzer C, Brimmer A, Hermanns P, Zabel B, Dietz UH (2007) Expression profile of Papss2 (3′-phosphoadenosine 5′-phosphosulfate synthase 2) during cartilage formation and skeletal development in the mouse embryo. Dev Dyn 236: 1313–1318. 23. Leonova EV, Fairfield DA, Lomax MI, Altschuler RA (2002) Constitutive expression of Hsp27 in the rat cochlea. Hear Res 163: 61–70. 24. Groigno L, Richard-Parpaillon L, Boujard D (1999) Expression pattern of insulin receptor mRNA during Xenopus laevis embryogenesis. Mech Dev 86: 151–154. 25. Koyama M, Spicer SS, Schulte BA (1999) Immunohistochemical localization of Ca2+/Calmodulin-dependent protein kinase IV in outer hair cells. J Histochem Cytochem 47: 7–12. 26. Bok J, Wang Q, Huang J, Green SH (2007) CaMKII and CaMKIV mediate distinct prosurvival signaling pathways in response to depolarization in neurons. Mol Cell Neurosci 36: 13–26. 27. Iino Y, Kakizaki K, Katano H, Saigusa H, Kanegasaki S (2005) Eosinophil chemoattractants in the middle ear of patients with eosinophilic otitis media. Clin Exp Allergy 35: 1370–1376. 28. Goedken M, McCormick S, Leidal KG, Suzuki K, Kameoka Y, et al. (2007) Impact of two novel mutations on the structure and function of human myeloperoxidase. J Biol Chem 282: 27994–28003. 29. Watanabe K, Yagi T (2000) Expression of myeloperoxidase in the inner ear of cisplatin-treated guinea pigs. Anticancer Drugs 11: 727–730. 30. Wang IC, Chen YJ, Hughes D, Petrovic V, Major ML, et al. (2005) Forkhead box M1 regulates the transcriptional network of genes essential for mitotic progression and genes encoding the SCF (Skp2-Cks1) ubiquitin ligase. Mol Cell Biol 25: 10875–10894. 31. Zhang H, Ackermann AM, Gusarova GA, Lowe D, Feng X, et al. (2006) The FoxM1 transcription factor is required to maintain pancreatic beta-cell mass. Mol Endocrinol 20: 1853–1866. 32. Tan Y, Raychaudhuri P, Costa RH (2007) Chk2 mediates stabilization of the FoxM1 transcription factor to stimulate expression of DNA repair genes. Mol Cell Biol 27: 1007–1016. 33. Pauley S, Lai E, Fritzsch B (2006) Foxg1 is required for morphogenesis and histogenesis of the mammalian inner ear. Dev Dyn 235: 2470–2482. 34. Ruchaud S, Carmena M, Earnshaw WC (2007) Chromosomal passengers: conducting cell division. Nat Rev Mol Cell Biol 8: 798–812.
(0.21 MB DOC)
