# Supplementary figures and images for: RNA Microarray Analysis in Prenatal Mouse Cochlea Reveals Novel IGF-I Target Genes: Implication of MEF2 and FOXM1 Transcription Factors
Source: PLoS One. 2010 Jan 25;5(1):e8699. doi: 10.1371/journal.pone.0008699 (PMC2810322; doi:10.1371/journal.pone.0008699)

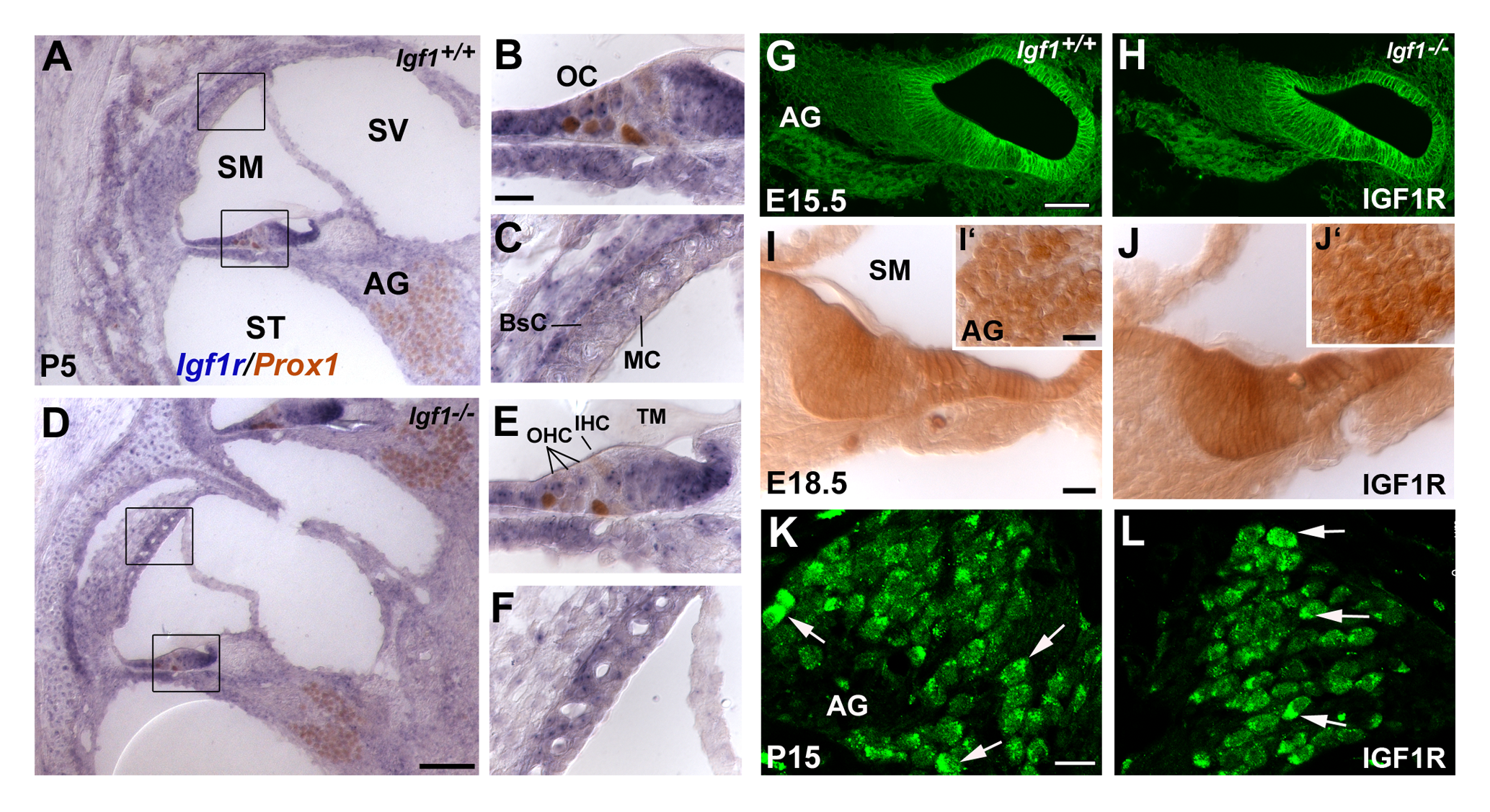

Supplement: Figure S1 — Spatiotemporal expression patterns of Igf1r mRNA and IGF1R protein in the Igf1+/+and Igf1−/− mouse cochlea. (A–F) shows that the mRNA expression of Igf1r was identical in the two genotypes. (A,B,C) P5 Igf1+/+ and (D,E,F) P5 Igf1−/− mice. IGF1R protein was shown at the organ of Corti and in the auditory ganglion at E15.5 (G,H) and E18.5 (I,I',J,J') with similar cellular localization between genotypes. At P15, the expression was located mainly in the neurons of the auditory ganglion (K,L), no differences could be observed. AG, auditory ganglion; BsC, basal cells IHC, inner hair cells; MC, marginal cells; OC, organ of Corti; OHC, outer hair cells; SM, scale media; ST, scala tympani; SV, scala vestibule, TM, tectorial membrane. Scale bars: A, 150 µm (A,D); B, 20 µm (B,C,E,F); G, 20 µm (G,H); I, 100 µm (I,J); I', 20 µm (I',J'); K, 20 µm (K,L). (3.53 MB DOC) [file pone.0008699.s001.tif]

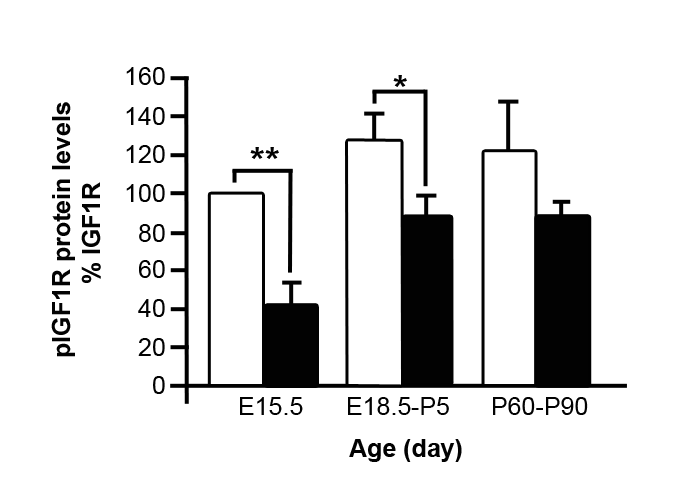

Supplement: Figure S2 — IGF-I deficiency modifies IGF1R phosphorylation levels.Protein extracts obtained from E15.5, E18.5, P5, P60 and P90 Igf1+/+ or Igf1−/− mouse cochleas (n = 6, from at least 2 different experiments) were immunoblotted to detect the presence of pIGF1R. Blots were re-probed with IGF1R as loading control. The specific bands were measured by densitometry (ImageJ software) to determine the average expression. Results were normalized, a value of 100 was assigned to the scanned intensity of the E15.5 Igf1+/+ cochlear extract. Statistical significance estimated with Student's t-test was: **p<0.01; *p<0.05. Open bars: Igf1+/+ mouse; Closed bars: Igf1−/− mouse. (0.05 MB TIF) [file pone.0008699.s002.tif]

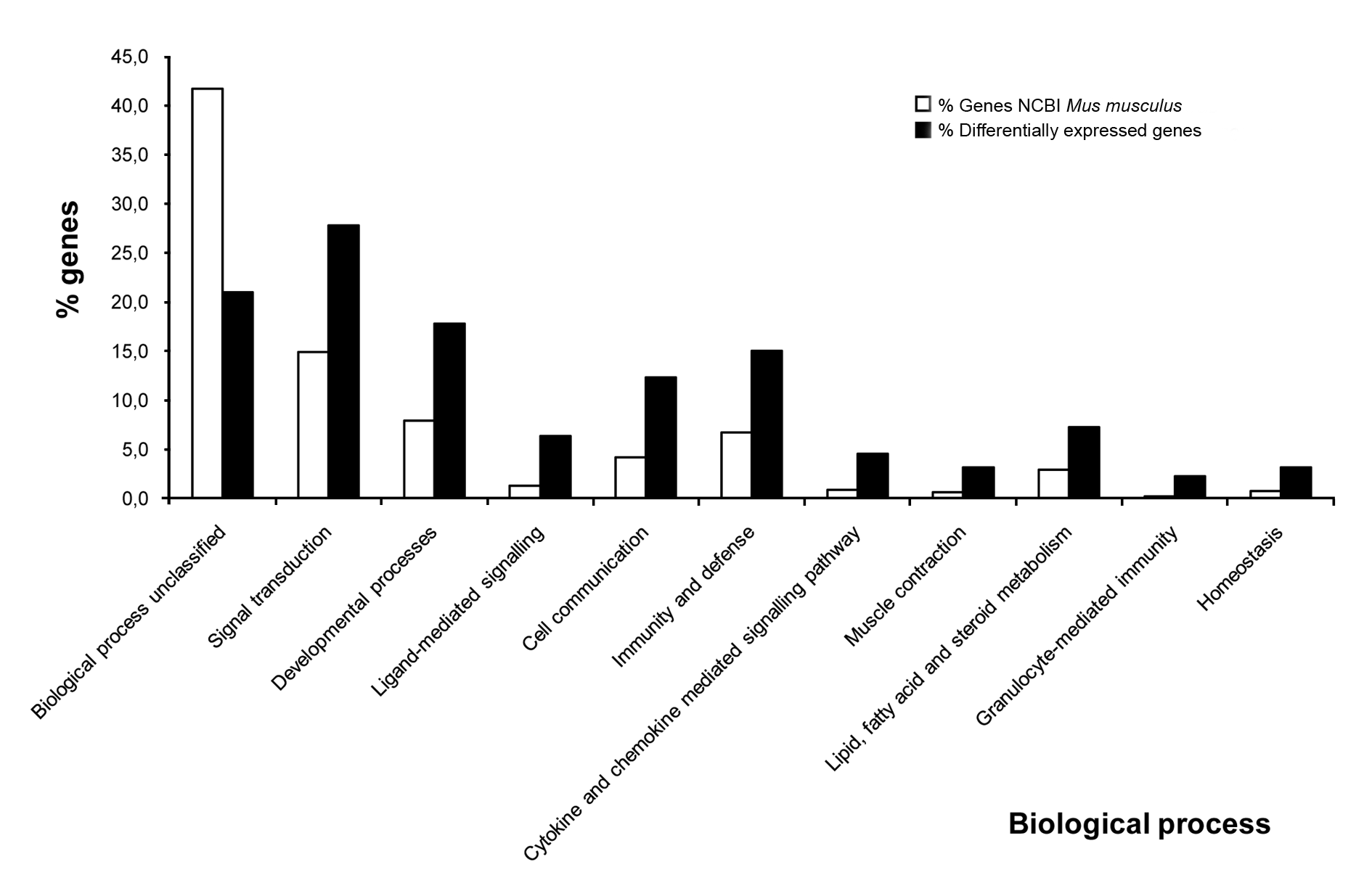

Supplement: Figure S3 — Clustering of the differentially expressed genes in Igf1−/− cochleas grouped according to their functional category. The 231 genes differentially expressed (closed bars), were classified by their biological annotation compared with all Mus musculus genome annotations in the NCBI (open bars). The statistical analysis of the biological processes included a Bonferroni correction for multiple testing and processes were selected at p<0.05. The differentially expressed genes are implicated in the following biological processes: signal transduction, developmental process, ligand-mediated signalling, cell communication, immunity and defence, cytokine and chemokine mediated signalling, muscle contraction, lipid, fatty acid and steroid metabolism, granulocyte-mediated immunity and homeostasis. (0.20 MB TIF) [file pone.0008699.s003.tif]

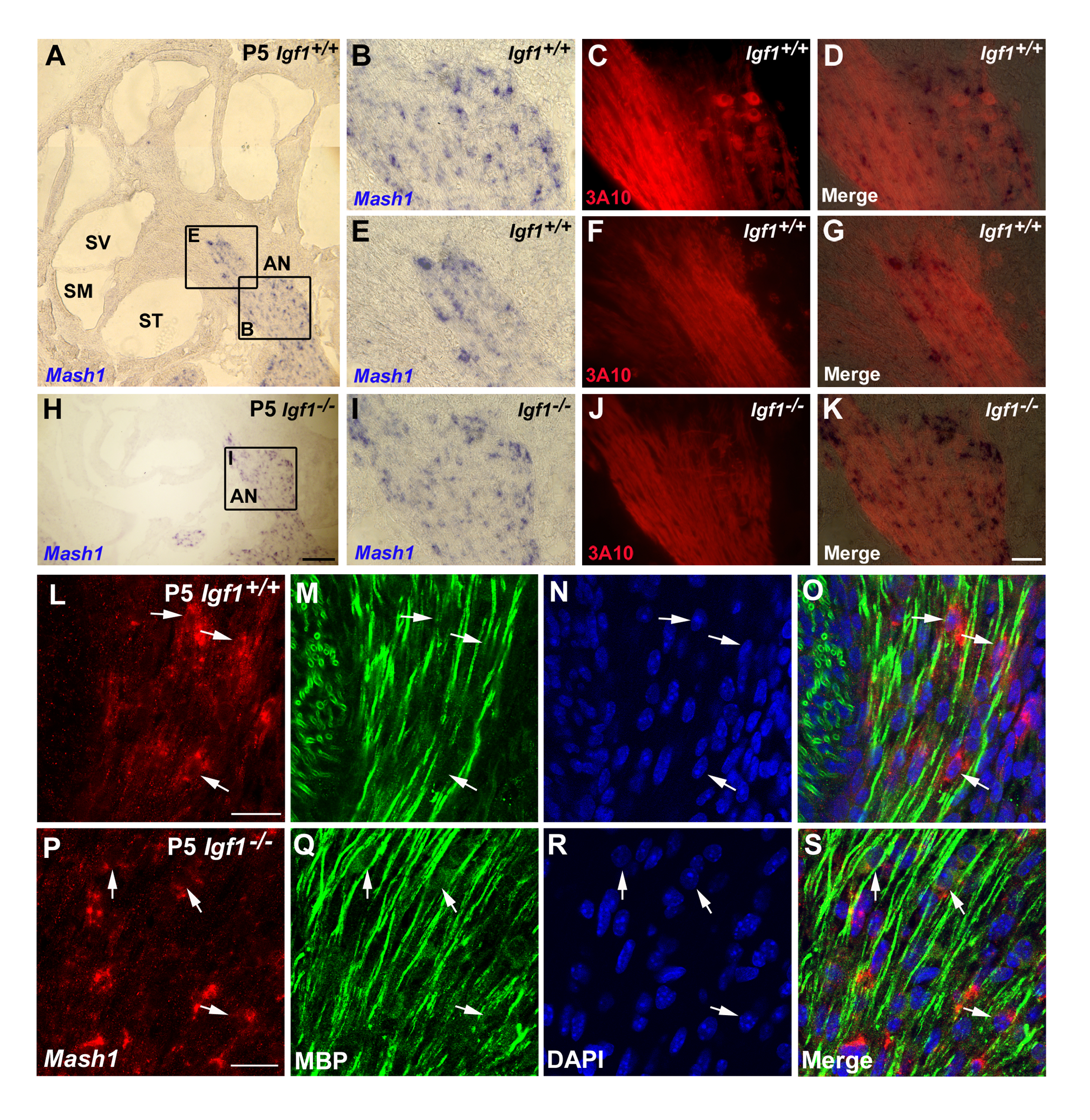

Supplement: Figure S4 — Mash1 expression in the Igf1+/+and Igf1−/− P5 mouse auditory nerve.(A–K) Mash1 in situ hybridization was performed on cryostat sections in Igf1+/+ (A–G) and Igf1−/− (H–K) cochleas at P5. Axons in the auditory nerve (AN) were recognised by 3A10 immunostaining (red C,D,F,G,J,K). At P5, Mash1 expression in the auditory nerve did not show differences between genotypes (B–G,I–K). B, E and I are higher magnification images of the boxed areas in A and H, respectively. D,G,K are merge images of Mash1 and 3A10 labelling. (L–S) Double in situ hybridization of Mash1 (red; L,P,O,S) and inmunohistochemistry of myelin basic protein (green, MBP; M,Q,O,S). Cell nuclei were stained with DAPI (blue; N,R,O,S). Mash1 expression was perinuclear and was associated at P5 to the soma of cells, probably root neurones, embedded in auditory axons (white arrows; L–S). Three embryos per genotype were tested in parallel in three independent experiments. AN, auditory nerve; SM, scala media; ST, scala tympani; SV, scala vestibuli. Scale bars: H, 150 µm (A, H); K, 30 µm (B–G,I–K), L, 35 µm (L–O); P, 20 µm (P–S). (7.24 MB TIF) [file pone.0008699.s004.tif]
