# Supplementary material for: RNA Microarray Analysis in Prenatal Mouse Cochlea Reveals Novel IGF-I Target Genes: Implication of MEF2 and FOXM1 Transcription Factors
Source: PLoS One. 2010 Jan 25;5(1):e8699. doi: 10.1371/journal.pone.0008699 (PMC2810322; doi:10.1371/journal.pone.0008699)
Supplement: Table S1 — Summary of inventoried TaqMan probes used for qRT-PCR. Applied Biosystems: https://products.appliedbiosystems.com/ab/en/US/adirect/ab?cmd=ABGEKeywordSearch. (0.06 MB DOC) [file pone.0008699.s005.doc]

| **Gen symbol** | **TaqMan probes** |
| --- | --- |
| Akr1c13 | Akr1c13-Mm00657347_m1 |
| Cacna1f | Cacna1f-Mm00490443_m1 |
| Cldn18 | Cldn18-Mm00517320_m1 |
| Cntn2 | Cntn2-Mm00516138_m1 |
| Dnajb7 | Dnajb7-Mm00498649_s1 |
| Fgf15 | Fgf15-Mm00433278_m1 |
| Fibp | Fibp-Mm00517555_m1 |
| Foxg1 | Foxg1-Mm02059886_s1 |
| Foxm1 | Foxm1-Mm 00514924_m1 |
| Igf1 | Igf1-Mm00439561_m1 |
| Igf1r | Igf1r-Mm00802831_m1 |
| Igfbp2 | Igfbp2-Mm00492632_m1 |
| Igfbp3 | Igfbp3-Mm00515156_m1 |
| Ins2 | Ins2-Mm00731595_gH |
| Irs2 | Irs2-Mm03038438_m1 |
| Kcnd2 | Kcnd2-Mm00498065_m1 |
| Kif17 | Kif17-Mm00456740_m1 |
| Mash1 | Mash1-Mm03058063_m1 |
| Mef2a | Mef2a-Mm01318991_m1 |
| Mef2c | Mef2c- Mm00600423_m1 |
| Mef2d | Mef2d-Mm00504931_m1 |
| Mpz | Mpz-Mm00485139_m1 |
| Myo7a | Myo7a-Mm00485371_m1 |
| Nes | Nes-Mm00450205_m1 |
| Retnla | Retnla-Mm00445109_m1 |
| Rp1h | Rp1h-Mm00803370_m1 |
| S100g | S100g-Mm00486654_m1 |
| Scgb1a1 | Scgb1a1-Mm00442046_m1 |
| Shbg | Shbg-Mm00839856_g1 |
| Six6 | Six6-Mm00488257_m1 |
| Slc19a2 | Slc19a2-Mm00499760_m1 |
| Syp | Syp-Mm00436850_m1 |
| Trap1a | Trap1a-Mm00495785_m1 |
| Tub | Tub-Mm00499015_m1 |
| Ush1c | Ush1c-Mm00458359_m1 |
| Uts2r | Uts2r-Mm00520770_s1 |
| Vegfa | Vegfa-Mm00437304_m1 |
| Vim | Vim-Mm00449201_m1 |
| 18S | 18S-Hs99999901_s1 |
