# Supplementary material for: RNA Microarray Analysis in Prenatal Mouse Cochlea Reveals Novel IGF-I Target Genes: Implication of MEF2 and FOXM1 Transcription Factors
Source: PLoS One. 2010 Jan 25;5(1):e8699. doi: 10.1371/journal.pone.0008699 (PMC2810322; doi:10.1371/journal.pone.0008699)
Supplement: Table S2 — Summary of cDNAS used to generate the in situ hybridization probes. Prior to the in situ hybridization, all clones were sequenced (ABI 3130XL Applied Biosystems). At least 3 embryos per genotype were tested in parallel in three independent experiments. No signal was obtained with the sense probe (data not show) * Igf1r probe was the generous gift of Prof. Flora de Pablo (CIB, CSIC, Madrid) 1References 1. Lopez-Rios J, Gallardo ME, Rodriguez de Cordoba S, Bovolenta P (1999) Six9 (Optx2), a new member of the six gene family of transcription factors, is expressed at early stages of vertebrate ocular and pituitary development. Mech Dev 83: 155–159. 2. Guillemot F, Joyner AL (1993) Dynamic expression of the murine Achaete-Scute homologue Mash-1 in the developing nervous system. Mech Dev 42: 171–185. 3. McWhirter JR, Goulding M, Weiner JA, Chun J, Murre C (1997) A novel fibroblast growth factor gene expressed in the developing nervous system is a downstream target of the chimeric homeodomain oncoprotein E2A-Pbx1. Development 124: 3221–3232. 4. Hayashi T, Cunningham D, Bermingham-McDonogh O (2007) Loss of Fgfr3 leads to excess hair cell development in the mouse organ of Corti. Dev Dyn 236: 525–533. 5. Ladher RK, Wright TJ, Moon AM, Mansour SL, Schoenwolf GC (2005) FGF8 initiates inner ear induction in chick and mouse. Genes Dev 19: 603–613. 6. Liu JP, Baker J, Perkins AS, Robertson EJ, Efstratiadis A (1993) Mice carrying null mutations of the genes encoding insulin-like growth factor I (Igf-1) and type 1 IGF receptor (Igf1r). Cell 75: 59–72. (0.05 MB DOC) [file pone.0008699.s006.doc]

| **cDNA** | **Antisense probe** | **Sense probe** | **References** |
| --- | --- | --- | --- |
| ***Six6*** | EcoRI/T7 | HindIII/T3 | [1]^1^ |
| ***Mash1*** | Xba1/Sp6 | BsaI/T7 | [2] |
| ***Fgf15*** | Not1/T7 | NcoI/Sp6 | [3] |
| ***Fgfr3*** | Xho1/T3 | EcoR1/T3 | [4] |
| ***Fgf8*** | EcoRI/T3 | BamHI/T7 | [5] |
| ***Igf1*** | XbaI/T3 | HindIII/T7 | [6] |
| ***Igf1r*** | HindIII/T7 | NotI/Sp6 | de Pablo, F* |
