# Supplementary material for: RNA Microarray Analysis in Prenatal Mouse Cochlea Reveals Novel IGF-I Target Genes: Implication of MEF2 and FOXM1 Transcription Factors
Source: PLoS One. 2010 Jan 25;5(1):e8699. doi: 10.1371/journal.pone.0008699 (PMC2810322; doi:10.1371/journal.pone.0008699)
Supplement: Table S3 — Summary of antibodies used for immunohistochemistry and Western blotting. For IHC, at least 3 embryos per genotype were tested in parallel in three independent experiments. Control experiments without primary antibody were carried out for each reaction and indicated that the staining pattern was specific for antigen recognition (data not shown). 1Antibody type: RbP, rabbit polyclonal; MouM mouse monoclonal; GP, goat polyclonal Abbreviations: DSHB, Developmental Studies Hybridoma Bank; IHC, immunohistochemistry; WB, western blotting. (0.06 MB DOC) [file pone.0008699.s007.doc]

| *Antibody* | *Type^1^* | *Source/Cat #* | *Concentration* |
| --- | --- | --- | --- |
| **3A10** | MouM | DSHB/3A10 | 1:5 (IHC) |
| **β-actin** | MouM | Sigma/A5441 | 1:1000 (WB) |
| **Akt1/2** | GP | Santa Cruz/SC-1619 | 1:1000 (WB) |
| **phospho-Akt^Ser473^** | RbP | Cell Signalling/9271 | 1:1000 (WB) |
| **ERK MAPK** | RbP | Cell Signalling/9102 | 1:1000 (WB) |
| **phospho-ERK MAPK** | RbP | Cell Signalling/9101 | 1:1000 (WB) |
| **FoxM1** | RbP | Santa Cruz/SC-500 | 1:1000 (WB) |
| **histone H3** | MouM | Upstate/05-499 | 0.5μg/ml (WB) |
| **IGF-IR (Tyr 1165/1166)** | RbP | Santa Cruz/SC-101704 | 1:50 (IHC)/1:1000 (WB) |
| **IGF-IRβ** | RbP | Santa Cruz/SC-713 | 1:50 (IHC)/1:1000 (WB) |
| **IRS2** | RbP | Upstate/06-506 | 1:400 (IHC)/1:1000 (WB) |
| **Kir4.1** | RbP | Chemicon/AB5818 | 1:200 (IHC) |
| **MEF2A** | RbP | Santa Cruz/SC-313 | 1:50 (IHC)/1:1000 (WB) |
| **MEF2C** | GP | Santa Cruz/ SC13266 | 1:50 (IHC)/1:250 (WB) |
| **MEF2D** | MouM | BD Bioscience/610774 | 1:1000 (IHC)/1:1000 (WB) |
| **Myelin Basic Protein** | RbP | Biotrend/BT300-0082 | 1:200 (IHC) |
| **MyosinVIIa** | RbP | Proteus Biosciences/25-6790 | 1:150 (IHC) |
| **p27Kip1** | MouM | BD Bioscience/610241 | 1:2500 (WB) |
| **P38 MAP Kinase** | RbP | Cell Signalling/9212 | 1:1000 (WB) |
| **phospho-P38 MAPK** | MouM | Cell Signalling/9216 | 1:2000 (WB) |
| **p75** | RbP | Promega/G3223 | 1:500 (IHC) |
| **Prox1** | RbP | Chemicon/AB5475 | 1:1000 (IHC) |
