# Supplementary material for: RNA Microarray Analysis in Prenatal Mouse Cochlea Reveals Novel IGF-I Target Genes: Implication of MEF2 and FOXM1 Transcription Factors
Source: PLoS One. 2010 Jan 25;5(1):e8699. doi: 10.1371/journal.pone.0008699 (PMC2810322; doi:10.1371/journal.pone.0008699)
Supplement: Table S4 — Differentially expressed genes in the E18.5 cochlea of the Igf1−/− mutant mouse ranked by gene ontology and biological process annotations. Genes were selected according to reported inner ear expression, their relation with deafness, biological function determined with the PANTHER and FATIGO programmes, as well as fold-change and low-variance calculated with multi-mgMOS software. nf, not described previously in the literature; nd, not determined by real-time PCR 1Average fold change from microarray experiments. 2Fold change determined by real-time PCR. nd, not determined; nv, not verified 3References 1. Coppens AG, Kiss R, Heizmann CW, Schafer BW, Poncelet L (2001) Immunolocalization of the calcium binding S100A1, S100A5 and S100A6 proteins in the dog cochlea during postnatal development. Brain Res Dev Brain Res 126: 191–199. 2. Kitajiri SI, Furuse M, Morita K, Saishin-Kiuchi Y, Kido H, et al. (2004) Expression patterns of claudins, tight junction adhesion molecules, in the inner ear. Hear Res 187: 25–34. 3. Fritzsch B, Pauley S, Beisel KW (2006) Cells, molecules and morphogenesis: the making of the vertebrate ear. Brain Res 1091: 151–171. 4. Chu PJ, Rivera JF, Arnold DB (2006) A role for Kif17 in transport of Kv4.2. J Biol Chem 281: 365–373. 5. Sokolowski BH, Sakai Y, Harvey MC, Duzhyy DE (2004) Identification and localization of an arachidonic acid-sensitive potassium channel in the cochlea. J Neurosci 24: 6265–6276. 6. Adamson CL, Reid MA, Mo ZL, Bowne-English J, Davis RL (2002) Firing features and potassium channel content of murine spiral ganglion neurons vary with cochlear location. J Comp Neurol 447: 331–350. 7. Langer P, Grunder S, Rusch A (2003) Expression of Ca2+-activated BK channel mRNA and its splice variants in the rat cochlea. J Comp Neurol 455: 198–209. 8. Ruttiger L, Sausbier M, Zimmermann U, Winter H, Braig C, et al. (2004) Deletion of the Ca2+-activated potassium (BK) alpha-subunit but not the BKbeta1-subunit leads to progressive hearing loss. Pro [file pone.0008699.s008.doc]

| **GENE NAME** | **FC Arrays^1^** | **FC  qRT-PCR^2^** | **FUNCTION** | **EXPRESSION IN THE INNER EAR** |
| --- | --- | --- | --- | --- |
| **Calcium binding proteins** |  |  |  |  |
| *S100g (S100 calcium binding protein G) [CABP1; Calb3; Cabp9k;]* | -4,1 | -1,4 | Calcium binding protein. S100 family participates in microtubule assembly, neurite extension, cell cycle progression and cell proliferation. | S100 proteins are expressed in different structures of the inner ear of many species including human, monkey, rat and mouse [1]. |
| **Cell-cell adhesion** |  |  |  |  |
| *Cldn18 (Claudin 18)* | -6,0 | nd | Integral membrane protein. Forms tight junction strands in epithelial cells. | In adult mice, the protein is located in the organ of Corti, marginal cells of the stria vascularis, Reissner's membrane, spiral limbus and vestibular sensory epithelium [2]. |
| **Cell migration** |  |  |  |  |
| *Mash1 (Mammalian achaete-scute homolog 1) [Ascl1, Hash1]* | 2,9 | 2,4 | bHLH Transcription Factor. It is involved in the development of specific neural lineages in most regions of the CNS, and of several lineages in the PNS. This family is highly conserved through the evolution and is involved in hair cell differentiation, Atoh1, and in sensory neurons differentiation, Neurog1 and Neurod1 [3]. | nf |
| **Insulin resistance** |  |  |  |  |
| *Retnla (Resistin like alpha) [Fizz1]* | -4,7 | -2,3 | Cytokine. Mitogenic, angiogenic, and vasoconstrictive effects. | nf |
| **Ion trasport/Ion channels** |  |  |  |  |
| *Cacna1f (calcium channel, voltage-dependent, alpha 1F subunit)* | -2,1 | -1,5 | Voltage-sensitive calcium channel. Mediates the entry of Ca^2+^ into excitable cells and it is also involved in a variety of calcium-dependent processes, including muscle contraction, hormone or neurotransmitter release, gene expression, cell motility, cell division and cell death. | nf |
| *Kcnd2 (potassium voltage-gated channel, Shal-related family, member 2) [Kv4.2]* | -1,3 | -1,0 | Pore-forming (alpha) subunit of voltage-gated rapidly inactivating A-type potassium channels. It is transported by Kif17 [4]. | Kv4.2 gene and protein expression has been found in the auditory hair cells of P15 chicken [5]. In mouse, apex neurons had a preponderance of Kv4.2 subunits, whereas base neurons possessed greater levels of KCa, Kv1.1, and Kv3.1 subunits [6]. |
| *Kcnmb1 (potassium large conductance calcium-activated channel, subfamily M, beta member 1) [BKb1]* | -1,1 | nd | Voltage-activated K(+) channels are important for shaping the receptor potentials of cochlear hair cells. | BK-type beta subunits are expressed strong in the IHC and weaker in the OHC. BK channels underlie the large K(+) conductance in IHC of mammals. BKb1-/- mice present normal hearing function and cochlear structure [7,8]. |
| *Mlc1 (megalencephalic leukoencephalopathy with subcortical cysts 1 homolog (human))* | 1,3 | nd | Membrane protein of unknown function with low homology to potassium channels. MLC1 is the first gene responsible for Megalencephalic leukoencephalopathy with subcortical cysts. This disease is an autosomal recessive neurological disorder in children characterized clinically by macrocephaly, deterioration in motor functions, cerebellar ataxia and mental decline. | In the mouse, MLC1 showed post-synaptic localization on the IHC and in afferent fibers of the IHC in the organ of Corti as evidenced by co-localization with calretinin. MLC1 was strongly expressed in the SG in non-myelinated and myelinated parts of the auditory nerve. In the SG, only very few neuronal cell bodies were MLC1-positive [9]. |
| **Lipid metabolism** |  |  |  |  |
| *Alox12 (arachidonate 12-lipoxygenase)* | -1,8 | nd | Oxygenase and 14,15-leukotriene A4 synthase activity. Belongs to the lipoxygenase family. | Its expression is increased in cells of the mouse Organ of Corti treated with cisplatinum [10]. |
| **Neurotransmitter transport and metabolism** |  |  |  |  |
| *Slc5a7 (solute carrier family 5 [choline transporter], member 7) [Cht1]* | -2,2 | nd | High affinity choline transporter involved in the synthesis of acetylcholine. | *Slc5a7* is expressed in efferents terminals of the IHC and OHC in adult rat [11]. |
| *Slc18a3 (solute carrier family 18 (vesicular monoamine), member 3) [VAT; VAChT]* | -1,7 | nd | Participates in acetylcholine transport in synaptic vesicles. | In the adult rat, it is expressed in efferent terminals of the IHC and OHC [11]. |
| **Organ development** |  |  |  |  |
| *Fgf15 (Fibroblast growth factor 15) [Orthologue of chicken and human Fgf19]* | 3,6 | 1,0 | Growth Factor. Regulates cell division and patterning within specific regions of the embryonic brain, spinal cord and sensory organs. Is an early otic placode inducer [12]. | In the developing chicken, it is expressed in the sensory epithelium and neurons of the cochlear-vestibular ganglion [13]. |
| **Perception of sound and light** |  |  |  |  |
| *Six6 (sine oculis-related homeobox 6 homolog [Drosophila])* | 2,9 | 5,8 | Transcription Factor. It is involved in eye development. *Six6*, in association with *Dach* corepressors, regulates proliferation by directly repressing cyclin-dependent kinase inhibitors, including the p27Kip1 promoter. | *The Six-1-/-* null mouse presents alterations in inner ear early development [14]. |
| *Tub (tubby candidate gene)* | -2,3 | nv | Membrane-bound transcription regulator that translocates to the nucleus in response to G-protein activation-dependent phosphoinositide hydrolysis. | The homozygous Tubby (tub/tub) mutant mouse presents an early progressive hearing loss and photoreceptor degeneration[15]. These alterations are similar to those reported for Usher type I patients. |
| *Rp1h (retinitis pigmentosa 1 homolog [human])* | 2,1 | 3,0 | Photoreceptor specific protein. Mutations in human RP1 are a common cause of dominant retinitis pigmentosa. The human Usher syndrome is an inherited condition characterized by hearing impairment and progressive vision loss. | nf |
| *Ush1c (Usher syndrome 1C homolog [human]) [Harmonin]* | 1,4 | 0,7 | Stereocillia protein. Harmonin interacts with cadherin 23 and myosine VIIA in growing stereocilia of the inner ear, to shape the functional stereocilia bundle. Mutations in the Harmonin gene cause Usher syndrome type I subtype C, characterized by hearing impairment and retinitis pigmentosa [16]. | Human and mouse stereocillia of sensory hair cells express Harmonin [16]. |
| *Rorb (RAR-related orphan receptor beta)* | 1,66 | nd | Transcription Factor. ROR-beta is such an orphan nuclear receptor, forming a subfamily with the closely related nuclear receptors ROR-alpha. Rorb -/- mice are blind, yet their circadian activity rhythm is still entrained by light-dark cycles (Andre et al., 1998) | nf |
| **Receptor-linked signal transduction** |  |  |  |  |
| *Fibp (Fibroblast growth factor [acidic] intracellular binding protein)* | -3,5 | -0,7 | Fibroblast growth factor binding protein. | nf |
| **Vesicle-mediated transport** |  |  |  |  |
| *Kif17 (Kinesin family member 17)* | -2,9 | -0,9 | Motor protein. Transports vesicles containing NMDA receptor 2B along microtubules. It has a role in the transport of Kv4.2. | nf |
| *Vamp1 (vesicle-associated membrane protein 1)* | -2,0 | nd | Membrane protein. Involved in the targeting and/or fusion of transport vesicles to their target membrane. It is involved in vesicle fusion at both poles of the cell [17]. | Vamp1, is present in hair cells and efferents fibers in the organ of Corti of guinea pig. |
| **Vitamin transport** |  |  |  |  |
| *Slc19a2 (solute carrier family 19 [thiamine transporter], member 2)* | -1,4 | -0,7 | High-affinity thiamine transporter. *Slc19a2* mutations underlie the clinical syndrome known as thiamine-responsive megaloblastic anemia characterized by anemia, diabetes, and sensorineural hearing loss. | Selective expression in IHC, which are lost in the *Slc19a2-/-* null mouse [18]. |
| **Xenobiotic metabolism** |  |  |  |  |
| *Akr1c13 (Aldo-keto reductase family 1, member C13)* | 3,1 | 1,3 | Reductase. Catalyzes the reduction of gluconic acid derivatives. | In a cochlear microarray study of the *sh2/sh2 null* mouse this gene was related to age progressive hearing loss [19]. |
| **Regulation of transcription** |  |  |  |  |
| *Esrrb (estrogen related receptor, betav)* | -1,9 | nd | Orphan nuclear receptors. Closely related to the estrogen receptors (ERs). ERRs bound to estrogen response elements and interfered in the ER signal pathway. | Is expressed and controls the development of the endolymph-producing cells of the inner ear: the strial marginal cells in the cochlea and the vestibular dark cells in the ampulla and utricle. Mutations of ESRRB cause autosomal-recessive non syndromic hearing impairment DFNB35 [20,21]. |
| **Sulfate metabolism** |  |  |  |  |
| *Papss2 (3'-phosphoadenosine 5'-phosphosulfate synthase 2)* | -1 | nd | Bifunctional enzyme with both ATP sulfurylase and APS kinase activity. In mammals, PAPS is the sole source of sulfate; APS appears to be only an intermediate in the sulfate- activation pathway. May have a important role in skeletogenesis during postnatal growth. | It is expressed in the otic vesicle at E12.5 in the mouse [22]. |
| **Response to stress** |  |  |  |  |
| *Hspb1 (heat shock protein 1) [Hsp27]* | -1,1 | nd | Stress-inducible molecular chaperone and regulator of actin polymerization. | In the rat, Hsp27 staining is localized to the cuticular plate and lateral wall of OHC. Hsp27-like immunostaining is also found in tension fibroblasts, in the root cells of the spiral limbus and in Reissner's membrane. The presence of Hsp27 in the actin-rich tension fibroblasts and OHC suggests a potential role in the regulation and maintenance of the actin cytoskeleton in these cells. The presence of high levels of constitutive Hsp27 may also provide a mechanism for pre-protecting these cells against environmental stressors [23]. |
| **Insulin receptor signalling pathway** |  |  |  |  |
| *Insr(insulin receptor)* | -1,1 | nd | Insulin receptor is a tetramer of 2 alpha and 2 beta subunits. The alpha and beta subunits are coded by a single gene and are joined by disulfide bonds, a mechanism parallel to that of its ligand, insulin | Xenopus: In situ hybridization analysis shows that InsR expression is restricted to regions of ectodermal and mesodermal origin, notably the encephalon, otic vesicles, optic vesicles, gills, somites and the pronephros [24]. |
| **Signal transduction** |  |  |  |  |
| *Camk4 (calcium/calmodulin-dependent protein kinase IV)* | -1,3 | nd | CaMKIV-CREB pathway is crucial for osteoclast differentiation and function. | In sections of the adult gerbil inner ear, moderate to strong immunoreactivity for Camk4 was present along the lateral borders of OHCs [25] and in the SG [26]. |
| **Inmune response/ inflammatory response** |  |  |  |  |
| *Ccl11 (small chemokine (C-C motif) ligand 11) [Scya11; eotaxin ]* | -1,5 | nd | Chemokine. In response to the presence of allergens, this protein directly promotes the accumulation of eosinophils (a prominent feature of allergic inflammatory reactions), but not lymphocytes, macrophages or neutrophils. | In the eosinophilic otitis media (EOM), chemokines such as ecalectin and eotaxin are also produced in the middle ear [27]. |
| *Il13ra1 (interleukin 13 receptor, alpha 1)* | 1,9 | nd | Cytokine receptor activity. Together with IL4R-alpha form a heterodimer functional receptor for IL13 and IL4 and is thought to be the main route by which nonhematopoietic cells respond to these cytokines. | nf |
| **Response to oxidative stress** |  |  |  |  |
| *Mpo (myeloperoxidase)* | 1,2 | nd | Lysosomal hemoprotein located in the azurophilic granules of polymorphonuclear leukocytes and monocytes. In response to stimulation, MPO is activated into a transient intermediate with potent antimicrobial oxidizing abilities [28]. | MPO could be detected after 3 days of the application of cisplatin, in the lateral wall, the organ of Corti, supporting cells of the sensory epithelium and dark cells. These results suggest that MPO and reactive oxygen species are involved in the inner ear dysfunction after the application of cisplatin [29]. |
| **Cell Cycle progression** |  |  |  |  |
| *FoxM1 (Forkhead box M1)* | 1.5 | 1,2 | Transcription Factor. FoxM1 is essential for mitotic progression and for the transcriptional response during DNA damage/checkpoint signalling [30-32]. | nf |
| *FoxG1 (Forkhead box G1)* | -8.9·10^-5^ | 0.15 | Transcription Factor. The specific function of this gene has not yet been determined; however, is a strong candidate gene for determining forebrain size in vertebrates due to its role in the development of the telencephalon, where it promotes progenitor proliferation and suppresses premature neurogenesis. | FoxG1 mouse mutants showed vestibular and cochlear defects, between then a shortened cochlea with multiple rows of hair cells and supporting cells and the lack of horizontal crista [33] |
| *Incenp (Inner centromere protein)* | -2.2 | nd | INCENP is a member of the chromosomal passenger complex (CPC). INCENP appears be a scaffold that interacts with the three other members of the complex, Aurora B, Survivin and Borealin [34] | nf |
